# A method for the inference of cytokine interaction networks

**DOI:** 10.1371/journal.pcbi.1010112

**Published:** 2022-06-22

**Authors:** Joanneke E. Jansen, Dominik Aschenbrenner, Holm H. Uhlig, Mark C. Coles, Eamonn A. Gaffney

**Affiliations:** 1 Wolfson Centre for Mathematical Biology, Mathematical Institute, University of Oxford, Oxford, United Kingdom; 2 Translational Gastroenterology Unit, John Radcliffe Hospital, University of Oxford, Oxford, United Kingdom; 3 Kennedy Institute of Rheumatology, University of Oxford, Oxford, United Kingdom; 4 NIHR Oxford Biomedical Research Centre, John Radcliffe Hospital, University of Oxford, Oxford, United Kingdom; 5 Autoimmunity, Transplantation and Inflammation, Novartis Institutes for BioMedical Research, Novartis Pharma AG, Basel, Switzerland; 6 Department of Paediatrics, John Radcliffe Hospital, University of Oxford, Oxford, United Kingdom; University of Pittsburgh, UNITED STATES

## Abstract

Cell-cell communication is mediated by many soluble mediators, including over 40 cytokines. Cytokines, e.g. TNF, IL1*β*, IL5, IL6, IL12 and IL23, represent important therapeutic targets in immune-mediated inflammatory diseases (IMIDs), such as inflammatory bowel disease (IBD), psoriasis, asthma, rheumatoid and juvenile arthritis. The identification of cytokines that are causative drivers of, and not just associated with, inflammation is fundamental for selecting therapeutic targets that should be studied in clinical trials. As *in vitro* models of cytokine interactions provide a simplified framework to study complex *in vivo* interactions, and can easily be perturbed experimentally, they are key for identifying such targets. We present a method to extract a minimal, weighted cytokine interaction network, given *in vitro* data on the effects of the blockage of single cytokine receptors on the secretion rate of other cytokines. Existing biological network inference methods typically consider the correlation structure of the underlying dataset, but this can make them poorly suited for highly connected, non-linear cytokine interaction data. Our method uses ordinary differential equation systems to represent cytokine interactions, and efficiently computes the configuration with the lowest Akaike information criterion value for all possible network configurations. It enables us to study indirect cytokine interactions and quantify inhibition effects. The extracted network can also be used to predict the combined effects of inhibiting various cytokines simultaneously. The model equations can easily be adjusted to incorporate more complicated dynamics and accommodate temporal data. We validate our method using synthetic datasets and apply our method to an experimental dataset on the regulation of IL23, a cytokine with therapeutic relevance in psoriasis and IBD. We validate several model predictions against experimental data that were not used for model fitting. In summary, we present a novel method specifically designed to efficiently infer cytokine interaction networks from cytokine perturbation data in the context of IMIDs.

## Author summary

Cytokines are the messenger molecules of the immune system, allowing intercellular communication and mediating effective immune responses. They are an important therapeutic target in immune mediated inflammatory diseases such as inflammatory bowel disease (IBD) and rheumatoid arthritis. Cytokines interact in a tightly regulated network and depending on the context a particular cytokine can be involved in anti-inflammatory or inflammatory activities. In order to determine which cytokines to target in specific disease types and patient subsets, it is critical to study the effects of the inhibition of one or more cytokines on the larger cytokine interaction network. We present a novel method to extract a minimal, weighted network from cytokine interaction data. Existing biological network inference methods typically consider the correlation structure of the underlying dataset and/or make further assumptions of the dataset such as the existence of a small core of regulators. This can make them poorly suited for highly connected, non-linear cytokine interaction data. We validated our method using synthetic data and applied our method to a dataset on the regulation of IL23, a cytokine implicated in IBD pathogenesis. Predictions of the extracted IL23 network were validated using additional experimental data and were used to support the view of the cytokines IL1 and IL23 as promising targets for those patients that fail to respond to TNF*α* inhibition, the current golden standard in IBD treatment.

This is a *PLOS Computational Biology* Methods paper.

## Introduction

Inflammatory processes are tightly controlled by complex networks of cytokines. Immune cells and other cell types produce and secrete cytokines in response to a range of stimuli, such as pathogen-derived factors or cellular stress. Cytokines can act as stimuli themselves, either up-, or down-regulating further cytokine secretion. The dysregulation of cytokines is common to the wide array of immune mediated inflammatory diseases (IMIDS) such as inflammatory bowel disease (IBD), rheumatoid arthritis (RA), atherosclerosis, and Alzheimer’s disease. Inhibition of the pro-inflammatory cytokine TNF*α* has proven to be a very successful strategy in the treatment of RA, IBD, and other IMIDs. The success of TNF*α* inhibition has inspired the development of several other anti-cytokine therapies, such as anti-IL6(R) and anti-p_40_, the common subunit of IL12 and IL23 [1–4]. The success of cytokine therapy is disease specific. The inhibition of IL17A for instance aggravated Crohn’s disease, although it was effective in psoriasis and ankylosing spondylitis [5–8]. Further, various distinct mechanisms could be driving the pathogenesis of a particular IMID, requiring disease subtype specific treatment options. Around 10–30% of Crohn’s disease patients for instance do not respond to anti-TNF*α* therapy, and 23–46% of patients lose response over time [9]. These patients might benefit from the targeting of a different cytokine. In short, improving our understanding of the interactions of cytokines in various immunological contexts is critical for identifying new cytokine targets, and improving current treatment options in a disease (subtype) specific way. Such improvements in our understanding of cytokines also has extensive potential benefit in the rational development of cytokine representations within the large field of modelling inflammation processes, as exemplified by studies of tuberculosis infection [10], wound inflammation [11], neuroinflammation [12] and myocardial infarction [13].

**Fig 1:**
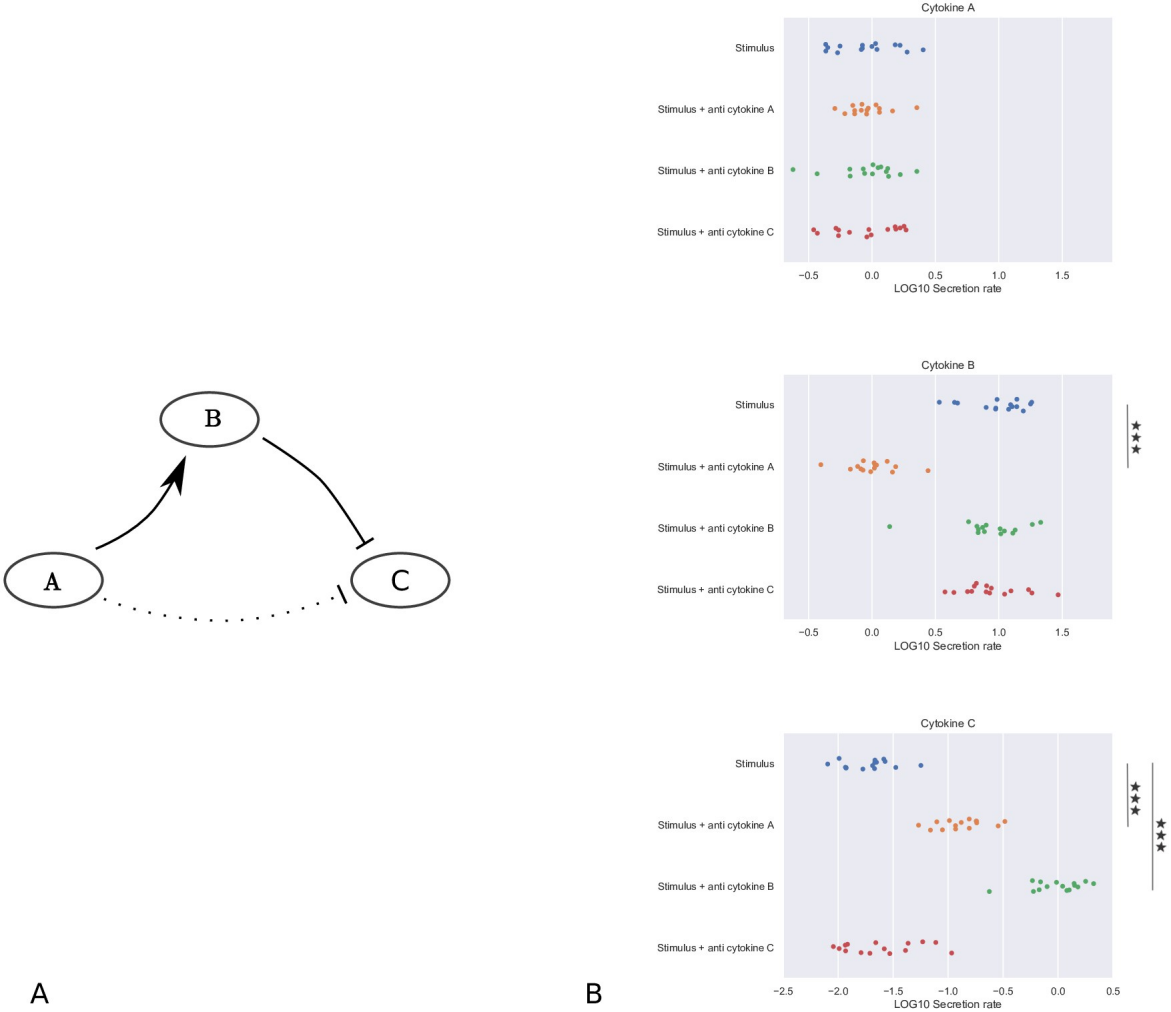
Example network motif and corresponding synthetic dataset. A: Addition of cytokine A up-regulates cytokine B secretion, whilst an increased concentration of cytokine B down-regulates cytokine C secretion. An increase in cytokine A concentration does not directly regulate cytokine C secretion (dotted edge), but only indirectly, via its effect on cytokine B secretion. B: Example synthetic dataset corresponding to the network motif shown in A. The dataset contains log-normally distributed synthetic cytokine secretion data from 15 ‘donors’ at a known moment in time, under the control condition (i.e. stimulus only, in blue), and in the presence of inhibitors of the cytokines of interest. For each of the four experimental conditions, cytokine secretion data for each of the three cytokines A, B and C is available. Full details on the construction of this dataset can be found in Section “Validation”. Using a two sample t-test, we test the hypothesis that the presence of inhibitor induces a change relative to the control: ^★★★^p≤ 0.001, false discovery rate (fdr) corrected.

To proceed, we define a cytokine interaction as the impact of one cytokine on the cytokine secretion rate of either itself or another cytokine. Identifying cytokine interactions, including key drivers of inflammation within the larger cytokine network, is an experimentally challenging task [14]. The blockage or addition of a cytokine can have both positive and negative effects on the secretion rate of other cytokines. However, only considering these pairwise interactions can be misleading, as cytokine networks are typically highly connected. Addition of one cytokine, “A”, could for instance up-regulate the secretion rate of another cytokine, “B”, whilst an increased cytokine B concentration down-regulates the secretion rate of a third cytokine, “C”. One might therefore conclude that an increased cytokine A concentration has a direct down-regulatory effect on cytokine C secretion, whilst this effect is completely dependent on its regulation of cytokine B secretion (Fig 1A).

We develop a method to extract a network of cytokine interactions from experimental data containing information on the effects of the blockade or addition of individual cytokines. Our method allows us to (1) distinguish between direct effects (e.g. cytokine A to cytokine B and cytokine B to cytokine C from the above example), and indirect effects (e.g. cytokine A to cytokine C), (2) quantify the size of the effects of cytokine inhibition, and (3) predict the effects of untested (combinations of) cytokine inhibitors. We use a simple coupled ODE system to represent cytokine interactions. We describe an algorithm to compute the optimal subset of network edges for each given number of edges. We use the Akaike information criterion (AIC) for model selection to identify the optimal network configuration [15, 16]. We have chosen an ODE approach over a discrete Boolean model, as an ODE model allows a quantification of cytokine input levels and secretion rates on a continuous scale [14, 17, 18]. Furthermore, this type of model could be extended to incorporate more complicated (non-linear) interactions than currently considered, and can be used to analyse data at various points in time. Our method requires data on the effects of the blockage of single cytokines on the system (interventional data). If such data is not available (i.e. if only observational data is available), other network inference methods may be used, such as Graphical Gaussian models or Bayesian networks [14, 19–23]. Approaches for network inference with other biological datasets, such as those associated with gene regulation or protein interactions, typically consider the correlation structure of the underlying dataset [22, 24] and/or make further assumptions of the dataset, such as the existence of a small core of regulators [25] or small numbers of pair-wise interactions [26], or a mutual exclusion of regulated and regulating entities [26, 27] that entails these studies and their techniques can be poorly suited for highly connected cytokine interaction data. As an example, such data ubiquitously has the exemplar scenario where one cytokine“A” (e.g. non-inflammatory IL10) might down-regulate cytokine“B” (e.g. pro-inflammatory IL1) secretion (negative correlation), but cytokine B might simultaneously upregulate cytokine A secretion (positive correlation), while both positive and negative self-edges cannot be identified by correlation based methods or methods where the regulators are distinct from the entity being regulated.

The study is structured as follows. After a Method section, we have a Section entitled “Validation” where the model is validated before being applied in Section “Application to a dataset on IL23 regulation”) to an experimental IL-23 dataset in the context of inflammatory bowel disease, with an experimental validation of the model predictions. We end with a discussion.

## Method

Based on a current experimental framework, as described in the Section on the “Application to a dataset on IL23 regulation”, our method requires an experimental dataset of cytokine secretion by *in vitro* stimulated immune cells in the presence and absence of various cytokine (receptor) inhibitors. We define an experimental condition as a specific combination of stimulus and cytokine (receptor) inhibitor. For each condition, a measurement *j* should contain single values for the secretion rate of each cytokine of interest at a known moment in time. We further assume we have multiple measurements for each condition, for instance corresponding to measurements obtained from cells from different donors, and the measurements for each condition and each cytokine, after a suitable scaling and taking its base 10 logarithm, follow a normal distribution, giving the form of the likelihood function, as we illustrate below.

This also raises the question of how to deal with a measurement dataset for a cytokine and experimental condition that does not successfully test as log-normally distributed even after scaling. In the example application to datasets from IL-23 regulation presented in Section (“Application to a dataset on IL-23 regulation”) the raw datasets for cytokine secretion under a given experimental condition are not generally log-normally distributed. Nonetheless, a donor-dependent scaling can rescale the datasets prior to taking base 10 logarithms to produce a log-normal distribution in all cases, as confirmed by statistical testing, enabling the use of the method below. More generally, in applying this method one must have sufficient experimental data to test and confirm the distribution of the dataset, or a scaling thereof. If the distribution is not log-normal the likelihood function used below is changed accordingly, with the rest of the method inherited. However, we restrict the synthetic data examples to log-normality, since log-normality is tested and confirmed for all the scaled experimental datasets that we have examined.

Proceeding, we assume the immune cells produce cytokines in response to the stimulatory agent used, such as the bacterial cell-wall component lipopolysaccharide (LPS), or the bacterial superantigen *Staphylococcus* enterotoxin B (SEB). We further assume that the secretion rate of certain cytokines is dependent on the concentration of other cytokines. In the simple example network motif discussed before (shown in Fig 1A), the secretion rate of cytokine B is dependent on the concentration of cytokine A, and the secretion rate of cytokine C is dependent on the concentration of cytokine B. In Fig 1B, we show an example dataset, corresponding to the example network motif shown in Fig 1A. This synthetic dataset contains log-normally distributed data from 15 ‘donors’, under the control condition (i.e. stimulus only), and in the presence of inhibitors of the cytokines of interest. Full details on the construction of this dataset will be presented in Section “Validation”.

We can represent all cytokines and their interactions by a network. Each node in this network represents a cytokine and each directed edge an up- or down-regulatory effect from one cytokine on another. Given a number of cytokine nodes, the aim of our method is to identify the network configuration, represented by a specific combination of directed edges, that optimally balances model complexity and fit. In particular, we aim to distinguish between direct and indirect interactions.

**Definition 1** (direct interaction). We define a direct cytokine interaction as a down- or upregulatory effect of the concentration of one cytokine (e.g. A) on the cytokine secretion rate of another cytokine (e.g. B), that is independent of the effects of the concentrations of other cytokines part of the studied network.

**Definition 2** (indirect interaction). We define an indirect cytokine interaction as a down- or upregulatory effect of the concentration of a cytokine (e.g. A) on the cytokine secretion rate of another cytokine (e.g. C), that is dependent of the effects of the concentrations of other cytokines part of the studied network.

We note that direct effects might be dependent on cytokines not present in the network. E.g. Cytokine A might upregulate cytokine Z that in turn upregulates cytokine B. If the secretion rate of cytokine Z is not measured and cytokine Z is therefore not represented by a node in the network, the effect of A on B (via Z) is direct, according to above definition. Cytokine Z might for instance be produced by lymphocytes, while only monocyte produced cytokines were measured. Conversely, if cytokine Z is part of the network, the cytokine Z-dependent effect of A on B is indirect. We introduce our model equations:

$$\begin{array}{cc} \frac{\text{d}x_{i}}{\text{d}t}=y_{i}\left(t\right)=s_{i}\prod_{u}(1+\alpha_{u,i}x_{u}\left(t\right))\prod_{v}(\frac{1}{1+\beta_{v,i}x_{v}\left(t\right)}),\;i,u,v=1,\ldots , & \left(1\right)\\ x_{i}\left(0\right)=0,\;i=1,\ldots , & \left(2\right) \end{array}$$

with *x*_*i*_(*t*) and *y*_*i*_(*t*) the time dependent concentration and secretion rate of cytokine *i*, *s*_*i*_ the size of the effect of the stimulatory agent, and *α*_*u*,*i*_ ≥ 0 (*β*_*v*,*i*_ ≥ 0) the size of the positive (negative) effect of cytokine *u* (*v*) on the secretion rate of cytokine *i*. Similarly, *x*_*u*_(*t*) and *x*_*v*_(*t*) are the concentrations of cytokine *u*, *v*. We take the unit of time *t* in hours. No cytokines are present at *t* = 0h, i.e. *x*_*i*_(0) = 0. For a given number of cytokines, our model Eq (1) can represent a specific network configuration, by fixing all *α*_*u*,*i*_, *β*_*v*,*i*_ that do not correspond to an edge in this configuration to zero. The network edges correspond to all non-zero *α*_*u*,*i*_ and *β*_*v*,*i*_. We assume a cytokine *w* cannot simultaneously have a positive and negative effect on cytokine *i*, i.e. when *α*_*w*,*i*_ > 0, we have *β*_*w*,*i*_ = 0, and when *β*_*w*,*i*_ > 0, we have *α*_*w*,*i*_ = 0. The weight of an edge is defined as the value of *α*_*u*,*i*_ or *β*_*v*,*i*_. Note that whilst the secretion rate of a particular cytokine might decrease over time due to increasing inhibition, the simulated cytokine concentrations themselves are monotonously increasing as we did not include degradation or absorption terms in Eq (1). Based on the motivating experimental protocol, we assume the secretion rate of the cytokines is so much higher than the degradation and absorption rates, that the latter can be ignored in our *in vitro* setting and for our timescale of interest. In particular, this assumption is supported by *in vitro* peripheral blood mononuclear cells’ (PBMC) cytokine concentration time courses of up to 80 hours after LPS stimulation, which indicate that cytokine concentrations are either still gradually increasing by 72 hours or, to within experimental resolution, have plateaued [28]. We note that in an *in vivo* setting, cytokine clearance should not be ignored, as the half-lifes of cytokines in vivo can be in the order of minutes, due to diffusion and other processes [29]. The experimental cytokine secretion data of cytokine *i* for measurement *j* is given by ${\hat{y}}_{i,j}$. Each *j* has been obtained from a single donor and under a single stimulatory condition. For each network configuration, we can fit the right hand-side of the system of Eq (1) to an experimental dataset by log-likelihood minimization, once log-normality has been tested for and confirmed. We minimise -2log(L(θ)) over our parameter vector ***θ***, where ***θ*** contains the model parameters *s*_*i*_, and all *α*_*u*,*i*_, *β*_*v*,*i*_ that are not fixed to zero for the network configuration of interest, and L(θ) is the likelihood function:

$$\begin{array}{c} L\left(\theta \right)=\prod_{i}\prod_{j}\frac{1}{\sqrt{2\pi \sigma_{i}^{2}}}\text{exp}(-\frac{{\left({\text{log}}_{10}\left(y_{i,j}\right)-{\text{log}}_{10}\left({\hat{y}}_{i,j}\right)\right)}^{2}}{2\sigma_{i}^{2}}), \end{array}$$ (3)

with *y*_*i*,*j*_ and ${\hat{y}}_{i,j}$ the value of the simulated and experimentally measured secretion rate of cytokine *i* for data point *j*, and *σ*_*i*_ the standard deviation of the log transformed measured secretion rate of cytokine *i* [30]. We assume a fixed standard deviation *σ*_*i*_ for each cytokine, that should be determined from the data. We note that when a dataset with multiple time-points would be available, a time-dependent *σ*_*i*_ could be tested for and used if appropriate.

In order to identify the network configuration with an optimal balance between edge number and model fit, we use the Akaike information criterion (AIC) for model selection. The AIC value of a network configuration can be computed as

$$\begin{array}{c} \text{AIC}=D+2p, \end{array}$$ (4)

with $D=\min_{\theta }-2\;\text{log}(L(\theta ))$, L(θ) the likelihood function as defined in Eq (3), and *p* the number of model parameters. We recall that each network edge is represented by one model parameter (*α*_*u*,*i*_ or *β*_*v*,*i*_). Setting an edge to zero corresponds to the removal of a parameter from the model. We can rank network configurations by comparing their relative AIC values.

In theory, one could calculate the AIC value of each possible network configuration for a dataset and select the network configuration with the lowest AIC value as the one that optimally balances model complexity and model fit. However, for a growing number of cytokines, this approach quickly becomes infeasible, as the number of possible network configurations is given by $3^{K^{2}}$, with *K* the number of cytokines, i.e. a positive, a negative, or no edge from cytokine *i* to cytokine *j* for every cytokine, including the case *i* = *j*. We will therefore constrain the collection of possible edges and corresponding network configurations as follows. We recall each dataset contains control data and data in the presence of the blocking antibody of a specific cytokine(receptor). Using a 2 sample t-test (*p* ≤ 0.05, false discovery rate (fdr, [31]) corrected), we determine whether a statistically significant difference exists in the secretion of the different cytokines, between the control data and the data in the presence of each blocking antibody. We will only consider network configurations consisting of subsets of edges corresponding to these statistically significant effects of one cytokine on another. The identification of the set of statistically significant effects is Step 1 of our method.

**Fig 2:**
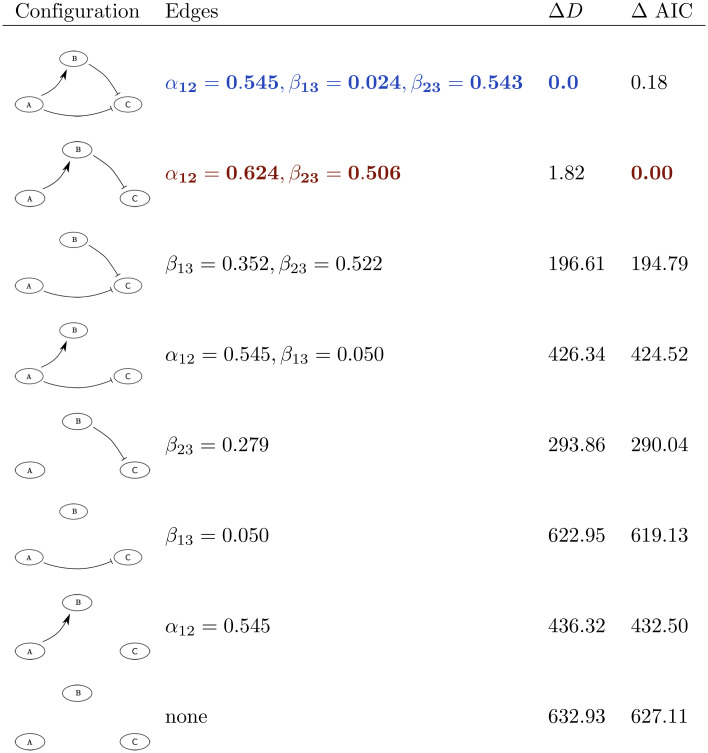
For each network configuration we list the model edges, the fitted edge parameter values, and the relative *D* and AIC when compared to their minimal found values, with $D=\min_{\theta }-2\;\text{log}(L(\theta ))$, L(θ) the likelihood function defined in Eq (3), and AIC defined in Eq (4). Whilst the three edge network (in blue) has the smallest relative *D*, the AIC selects the two edge network that was used to generate the synthetic dataset (in red). The edge parameter values used to generate this synthetic dataset are *α*_12_ = *β*_23_ = 0.5 and *β*_13_ = 0.

To illustrate this approach, we return to our example network motif and dataset (Fig 1). The total number of possible edges for this three node network is 18: a positive and negative edge to each of the network nodes for each cytokine. The ‘true’ number of edges, i.e. the number of edges of the model simulated to generate this synthetic dataset, is two: a negative effect from cytokine A to B, and a positive effect from cytokine B to C. For the shown synthetic dataset, the secretion rate of cytokines B and C is significantly higher in the presence of a cytokine A blocking antibody, and the secretion rate of cytokine C is significantly lower in the presence of a cytokine B blocking antibody, while no significant effect on any of the cytokine secretion rates is observed after addition of a cytokine C blocking antibody (Fig 1B). For our method we therefore consider the three network edges as shown in Fig 1A (i.e. A → B, B ⊣ C, and A ⊣ C), but not the 15 other possible network edges. Starting from the three edges, eight possible network configurations exist for this example, i.e. one configuration with zero edges, three configurations with one edge, three configurations with two edges, and one configuration with all three edges. We can select the one with the lowest AIC, given the data (see Fig 2, in red).

Now, we will describe how we can determine the network configuration with the lowest AIC, from all possible configurations of the set of statistically significant edges, without having to consider each of the possible sub-configurations individually. This is important, as the number of possible network configurations quickly becomes prohibitively large when the number of edges increases, even when only considering statistically significant edges. Let O be the set of *N* statistically significant edges. We denote network configurations by subsets $S^{n}\subseteq O$, with *n* the number of edges contained in the subset. We recall that each network configuration *S*^*n*^ corresponds to a model. The model size *n* corresponds to the number of edges contained in *S*^*n*^. Further, recall that the distance to the data for a given network configuration is defined by $D=\min_{\theta }-2\;\text{log}(L(\theta ))$, with the likelihood function L(θ) defined in Eq (3). We will refer to the distance to the data *D* for a network configuration *S*^*n*^ as *D*(*S*^*n*^).

**Definition 3** (Optimal *n*-sized model $S_{opt}^{n}$). We define the optimal model $S_{opt}^{n}$ for a given network size *n* as the model with the lowest distance *D* to the data, out of all *n*-sized sub-configurations of O, the set of *N* statistically significant edges.

**Remark 1.** When a smaller model is nested in a larger model, the distance between the experimental and simulated data of the smaller model has to be larger than or equal to the distance of the larger model, i.e. if *S*_*i*_ ⊂ *S*_*j*_, then *D*(*S*_*i*_) ≥ *D*(*S*_*j*_). This is the case because we can construct *S*_*j*_ by adding edges to *S*_*i*_. Each edge is represented by an edge parameter *α*_*u*,*v*_ ≥ 0 or *β*_*u*,*v*_ ≥ 0 in the model. Removal of an edge is equivalent to the fixation of the corresponding edge parameter to zero. When searching for a minimal *D*(*S*_*j*_) over all model parameters, this includes the case where the value of the edge parameters contained in *S*_*j*_ but not in *S*_*i*_ are all zero. The search space over all model parameters for a minimal *D*(*S*_*i*_) is therefore contained in the search space over all model parameters for a minimal *D*(*S*_*j*_). Therefore, *D*(*S*_*i*_) ≥ *D*(*S*_*j*_).

To find the network configuration with the lowest AIC, we will start from a network with all *N* statistically significant edges. We fit this network’s parameter values to the data and rank the edges monotonically in the value of the edge weight parameters for the *N*-edge graph. For instance with the simple test model we considered in Fig 2, the first ranked edge is the one connecting nodes 1 and 2, that is from A to B, since *α*_12_ = 0.545 for the *N* = 3 edge graph. This is then followed by the edge connecting nodes 2 and 3, that is from B to C, since *β*_23_ = 0.543, followed by the edge connecting nodes 1 and 3, that is from A to C since *β*_13_ = 0.024. With this ranking, we then generate a list of initial configurations $S_{init}^{n}$ for every network size *n* = 1, …, *N*, where each $S_{init}^{n}$ consists of the *n* edges corresponding to the *n* highest ranked fitted edge weight parameter values of the *N*-sized model. Thus, for the simple test model of Fig 2, the initial list would have the one edge, *n* = 1, network consisting of the edge connecting nodes 1 and 2, that is from A to B, since *α*_12_ = 0.545 is the largest fitted edge weight parameter, and hence $S_{init}^{1}=\left\{\alpha_{12}\right\}$. Similarly, the two edge, *n* = 2, network consists of the edge connecting nodes 1 and 2, that is from A to B since *α*_12_ = 0.545 is the largest fitted edge weight parameter together with the edge connecting nodes 2 and 3, that is from B to C since *β*_23_ = 0.543, is the second largest fitted edge weight parameter, so that $S_{init}^{2}=\{\alpha_{12},\beta_{23}$}. Similarly $S_{init}^{3}=\left\{\alpha_{12},\beta_{23},\beta_{13}\right\}$. We determine $D(S_{init}^{n})$ for every model configuration in the list. The generation of this initial list of network configurations is Step 2 of our method.

We note that apart from the *N*-edge network, for each *n*-sized model in the list, there might exist a different combination of edges resulting in a better fit to the data. Let AIC(*S*^*n*^) be the AIC value of model configuration *S*^*n*^, with *n* the number of edges. Recall that the AIC introduces a penalty of twice the number of added edges, i.e. when a model *S*^*n*^ of size *n* has a smaller AIC than a model *S*^*m*^ of size *m*, we have

$$\begin{array}{c} \text{AIC}\left(S^{n}\right)-\text{AIC}\left(S^{m}\right)=D\left(S^{n}\right)+2n-D\left(S^{m}\right)-2m<0. \end{array}$$ (5)

We define *S*_*selected*_ as the model configuration with the smallest AIC value out of all model configurations *S* for which we have determined *D*(*S*). At the start of Step 3 of our method, we have $S_{selected}=S_{init}^{n_{o}}$, with $n_{o}={\text{argmin}}_{n}\left[\text{AIC}\left(S_{init}^{n}\right)\right]$. For the simple test model we considered in Fig 2, we have $S_{selected}=S_{init}^{2}$ at the start of Step 3. For every model size *n*, we define

$$\begin{array}{c} D_{threshold}^{n}=D\left(S_{selected}\right)+2\left(n_{s}-n\right), \end{array}$$ (6)

with *n*_*s*_ the number of edges of *S*_*selected*_. We note that

**Remark 2**. If an *n*−sized model configuration *S*^*n*^ exists, with a lower or equal AIC than AIC(*S*_*selected*_), it follows that

$$\begin{array}{c} \text{AIC}\left(S_{selected}\right)-\text{AIC}\left(S^{n}\right)=D\left(S_{selected}\right)+2n_{s}-D\left(S^{n}\right)-2n=D_{threshold}^{n}-D\left(S^{n}\right)\geq 0, \end{array}$$

i.e. $D\left(S^{n}\right)\leq D_{threshold}^{n}$.

We describe a procedure (Procedure Ω) that we will use to update *S*_*selected*_ until *S*_*selected*_ equals the network configuration with the lowest AIC amongst all possible configurations of O, the set of *N* statistically significant edges. Procedure Ω takes input *S*_*selected*_ and a given model size *n*. For a given model size *n* and *S*_*selected*_, Procedure Ω identifies $S_{opt}^{n}$, but only if $D\left(S_{opt}^{n}\right)\leq D_{threshold}^{n}$, i.e. if $\text{AIC}\left(S_{opt}^{n}\right)\leq \text{AIC}\left(S_{selected}\right)$. When $D\left(S_{opt}^{n}\right)\leq D_{threshold}^{n}$, Procedure Ω returns an updated model configuration $S_{selected}=S_{opt}^{n}$. If it is found by Procedure Ω that no *n*-sized model configuration *S*^*n*^ exists, such that $D\left(S^{n}\right)\leq D_{threshold}^{n}$, it follows that $D\left(S_{opt}^{n}\right)>D_{threshold}^{n}$ and *S*_*selected*_ is not updated by Procedure Ω. Hence, after having run Procedure Ω for models of size *n*, we know that no *n*-sized models exist, with a smaller AIC than AIC(*S*_*selected*_). After having run Procedure Ω for model of all sizes *n* = 1, …, *N* − 1, we know that no models exist, with a smaller AIC than AIC(*S*_*selected*_). We call attention to the special case when Procedure Ω is run for models of size *n*_*s*_, with *n*_*s*_ the number of edges of *S*_*selected*_. Then, $D\left(S_{opt}^{n}\right)\leq D\left(S_{selected}\right)=D_{threshold}^{n}$ by Definition 3 and therefore Procedure Ω will always return the optimal model configuration $S_{opt}^{n_{s}}$ for models of size *n*_*s*_.

Before stating Procedure Ω, we first introduce the concept of an essential (combination of) edge(s):

**Definition 4** (Essential combination of edges). We call a non-empty subset of the initial configuration $S^{\prime }\subseteq S_{init}^{n}$ essential if $D\left(O\backslash S^{\prime }\right)\geq D_{threshold}^{n}$, where $O\backslash S^{\prime }$ is the model with all *N* statistically significant edges, except the edges contained in *S*′.

Further, we note that any *n*-sized model configuration *S*^*n*^ can be written in the form $S^{n}=R\cup \left(S_{init}^{n}\backslash S^{\prime }\right)$, with $S^{\prime }\subseteq S_{init}^{n}$ any subset of the initial configuration and $R\subseteq (O\backslash S_{init}^{n})$ a combination with the same number of edges as *S*′ (i.e. |*S*′| = |*R*|). To clarify, this means that any *n*-sized model consists of *l* edges that are part of $S_{init}^{n}$, and *n* − *l* edges that are not part of $S_{init}^{n}$, *l* = 0, 1, …, *n*. Therefore,

**Remark 3.** If there exists an *n*-sized model *S*^*n*^ such that $D\left(S^{n}\right)<D_{threshold}^{n}$, it has to be of the form $S^{n}=R\cup \left(S_{init}^{n}\backslash S^{\prime }\right)$, with $S^{\prime }\subseteq S_{init}^{n}$ any subset of the initial configuration and $R\subseteq (O\backslash S_{init}^{n})$ a combination with the same number of edges as *S*′ (i.e. |*S*′| = |*R*|).

Also,

**Lemma 1.** *If a combination of edges S*′ *is essential, no network configuration* $W\subseteq (O\backslash S^{\prime })$ *exists such that* $D\left(W\right)<D_{threshold}^{n}$ .

*Proof*. Let *S*′ be essential. It follows from the definition of an essential combination of edges that $D\left(O\backslash S^{\prime }\right)\geq D_{threshold}^{n}$. It then follows from Remark 1 that $D\left(W\right)\geq D\left(O\backslash S^{\prime }\right)\geq D_{threshold}^{n}$, with $W\subseteq (O\backslash S^{\prime })$.

**Lemma 2.** *If a combination of edges* $S^{\prime }\subseteq S_{init}^{n}$ *is essential, any combination of edges* $S^{\prime \prime }\subseteq S_{init}^{n}$ *such that S*′ ⊆ *S*′′ is also essential.

*Proof*. Let $S^{\prime }\subseteq S_{init}^{n}\subseteq O$ be essential and let $S^{\prime \prime }\subseteq S_{init}^{n}\subseteq O$ such that *S*′ ⊆ *S*′′. Then, $\left(O\backslash S^{\prime \prime }\right)\subseteq \left(O\backslash S^{\prime }\right)$. It follows from Remark 1 that $D\left(O\backslash S^{\prime \prime }\right)\geq D\left(O\backslash S^{\prime }\right)$. It then follows from the definition of an essential combination of edges that $D\left(O\backslash S^{\prime \prime }\right)\geq D\left(O\backslash S^{\prime }\right)\geq D_{threshold}^{n}$.

**Fig 3:**
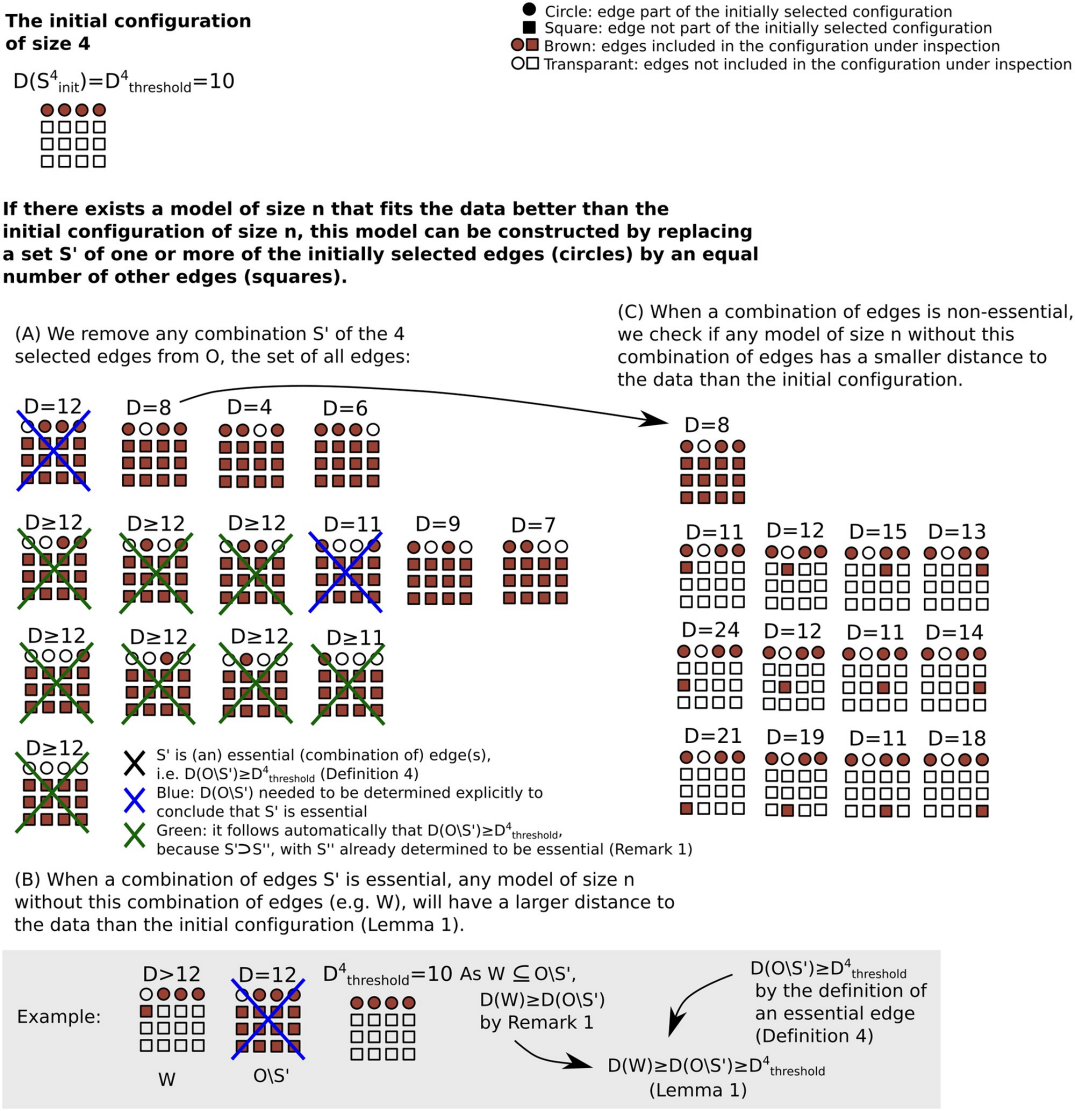
For the case $S_{selected}=S_{init}^{n}$, an overview of Procedure Ω to identify the optimal model, with the lowest distance *D* to the data, for a given model size *n* out of *N* statistically significant edges (SSEs). We show an example with initial configuration $S_{init}^{4}$ of size 4 and 16 SSEs. Circles represent edges in $S_{init}^{4}$ and squares represent edges that are not in $S_{init}^{4}$. We assume the model with the lowest AIC currently identified is $S_{init}^{4}$, i.e. $S_{selected}=S_{init}^{4}$, such that $D_{threshold}^{4}=D\left(S_{init}^{4}\right)=10$. We apply Procedure Ω to determine if a 4-sized model exists with a smaller *D* than $D(S_{init}^{4})$. If there exists a model *W* of size 4 that fits the data better than the initial configuration (i.e. *D*(*W*)<*D*_*threshold*_), this model can be constructed by replacing a set *S*′ of one or more of the initially selected edges (circles) by an equal number of other edges (squares). The brown fill colouring indicates edges included and the white fill correspond to edges excluded in the configuration under inspection. (A) We determine which of the 15 possible combinations of the 4 edges that are part of the initial configuration of size 4 are essential. We remove any combination *S*′ of the 4 selected edges from O, the set of all SSEs. When $D\left(O\backslash S^{\prime }\right)\geq D_{threshold}^{4}$, *S*′ is essential (Definition 4), which we indicate with a blue or green cross. Blue crosses correspond to configurations $O\backslash S^{\prime }$ for which we have to determine the value of $D(O\backslash S^{\prime })$ explicitly. Green crosses correspond to configurations $O\backslash S^{\prime }$, where *S*′ can be immediately deduced to be essential because *S*′′ contains an essential subconfiguration *S*′′ (Lemma 2). To clarify, consider a configuration of fifteen edges, excluding only a single edge from the initially selected model. When this configuration of 15 edges has a worse fit to the data than the initially selected model, removing further edges from this configuration will only reduce the quality of the fit and we thus do not have to determine their fit to the data explicitly (Remark 1). (B) We observe that if a subconfiguration *S*′ of edges that is part of the initial configuration $S_{init}^{n}$ is essential, then this subconfiguration *S*′ cannot be replaced in the initial configuration $S_{init}^{n}$ by any other combination of edges, e.g. *R*, with |*R*| = |*S*′|, in such a way that the resulting configuration $W=R\cup (S_{init}^{n}\backslash S^{\prime })$ fits the data better than the initial configuration *S*_*init*_ (grey insert). (C) If a subconfiguration *S*′ is not essential, we check if there exists another combination of edges, e.g. *R*, with |*R*| = |*S*′|, such that $R\cup (S_{init}^{n}\backslash S^{\prime })$ fits the data better than $S_{init}^{n}$.

In Fig 3 we give a graphical overview of the key steps of Procedure Ω, for *n* = 4, and with *N* = 16 statistically significant edges. For simplicity, we consider the special case where $S_{selected}=S_{init}^{n}$, i.e. the case where the model with the smallest AIC currently identified has size *n*. Procedure Ω then identifies the optimal model configuration $S_{opt}^{n}$ out of all model configurations of size *n*. In the general case, $D_{threshold}^{n}$ can be smaller than $D(S_{init}^{n})$, but the principle is the same. Note that Lemmas 1,2 are used extensively within Procedure Ω to reduce the number of networks that have to be explicitly considered, since the impact of Lemmas 1 and 2 is that any network that has the potential to generate an AIC improvement cannot exclude all the edges of an essential set of edges. In particular with a given fixed *n*, if $S^{\prime }\subseteq S_{init}^{n}$ is an essential set of edges, then excluding the edges of *S*′ from O, the set of *N* statistically significantly edges, entails by definition that the resulting distance from the data $D(O/S^{\prime })$ is greater than the threshold needed to improve the AIC score for any network constructed from the edges in $O/S^{\prime },$ including any of size *n*. In essence, we do not need to explicitly calculate the distance for any *n*-sized network with any set of essential edges excluded. Furthermore, for *n* fixed, any superset of a set of essential edges contained within $S_{init}^{n}$ is also essential by Lemma 2 (since more edges are excluded). Thus one need only perform the computationally relatively costly nested loop between steps 5–10 within Procedure Ω for subsets of edges that do not exclude all the edges of any essential set. Hence, the number of networks that need not be considered within steps 5–10 of Procedure Ω is extensive and thus there is a marked reduction in the computational demands required to identify the optimal network, as further detailed in the discussion.

**Fig 4:**
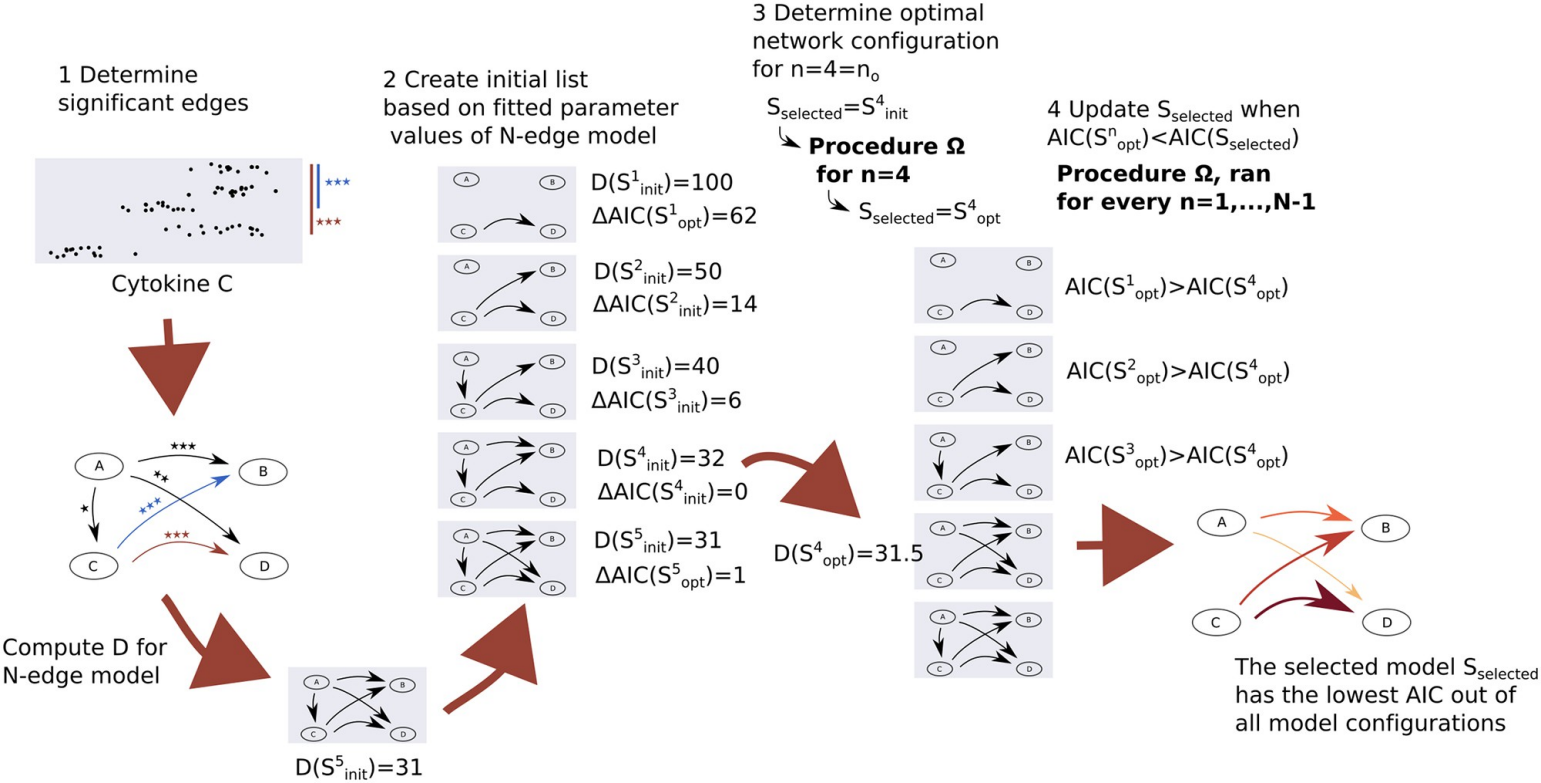
Overview of the key steps of our method. Our method requires log-normally distributed interventional cytokine secretion data that can be used to determine the value of D=-2log(L), with the likelihood L defined in Eq (3)), for each network configuration of interest. When the measurement data is not log-normally distributed, a donor-dependent scaling might in some cases be used to render the dataset to be log-normal (e.g. see Section “Application to a dataset on IL23 regulation”). We first (Step 1) perform a statistical analysis on the data to obtain a set of *N* statistically significant edges (2-sample t-test, *p* ≤ 0.05, fdr corrected). We then (Step 2) compute a list of initial configurations $S_{init}^{n}$ for each network size *n* ∈ {1, 2, …, *N*}. We fit the *N*-edge network to the data and let $S_{init}^{n}$ consists of the *n* edges corresponding to the *n* largest fitted edge parameter values of the *N*-sized model. We compute $D(S_{init}^{n})$ for each *n* ∈ {1, 2, …, *N*}. We define *S*_*selected*_ as the model configuration with the smallest AIC value currently identified. At the start of Step 3, we have $S_{selected}=S_{init}^{n_{o}}$, with $n_{o}={\text{argmin}}_{n}\left[\text{AIC}\left(S_{init}^{n}\right)\right]$. To identify the optimal *n*_*o*_-sized model configuration $S_{opt}^{n_{o}}$, we run Procedure Ω for models of size *n*_*o*_, with output $S_{selected}=S_{opt}^{n_{o}}$ (Step 3). We run Procedure Ω for models of size *n* = 1, 2, …, *N* − 1. Procedure Ω sets $S_{selected}=S_{opt}^{n}$ when $\text{AIC}\left(S_{opt}^{n}\right)\leq \text{AIC}\left(S_{selected}\right)$. After *N* runs of Procedure Ω, we have that *S*_*selected*_ has the smallest AIC of all possible subconfigurations of the set of statistically significant edges and corresponds to the model that best balances model complexity and fit.



We now state an overview of our method. Our method requires log-normally distributed interventional cytokine secretion data that can be used to determine the value of D=-2log(L), with the likelihood L defined in Eq (3)), for each network configuration of interest. When the measurement data is not log-normally distributed, a donor-dependent scaling might in some cases be used to rescale the dataset (e.g. see Section “Application to a dataset on IL23 regulation”), proceeding once log-normality is confirmed to within suitable tolerance. We first (Step 1) perform a statistical analysis on the data to obtain a set of *N* statistically significant edges (2-sample t-test, *p* ≤ 0.05, fdr corrected). We then (Step 2) compute a list of initial configurations $S_{init}^{n}$ for each network size *n* ∈ {1, 2, …, *N*}. We fit the *N*-edge network to the data and let $S_{init}^{n}$ consists of the *n* edges corresponding to the *n* largest fitted edge parameter values of the *N*-sized model. We compute $D(S_{init}^{n})$ for each *n* ∈ {1, 2, …, *N*}. We define *S*_*selected*_ as the model configuration with the smallest AIC value currently identified. At the start of Step 3, we have $S_{selected}=S_{init}^{n_{o}}$, with $n_{o}={\text{argmin}}_{n}\left[\text{AIC}\left(S_{init}^{n}\right)\right]$. To identify the optimal *n*_*o*_-sized model configuration $S_{opt}^{n_{o}}$, we run Procedure Ω for models of size *n*_*o*_, with output $S_{selected}=S_{opt}^{n_{o}}$ (Step 3). We run Procedure Ω for models of size *n* = 1, 2, …, *N* − 1. Procedure Ω sets $S_{selected}=S_{opt}^{n}$ when $\text{AIC}\left(S_{opt}^{n}\right)<\text{AIC}\left(S_{selected}\right)$. After *N* runs of Procedure Ω, we have that *S*_*selected*_ has the smallest AIC of all possible subconfigurations of the set of statistically significant edges and corresponds to the model that best balances model complexity and fit (Fig 4).

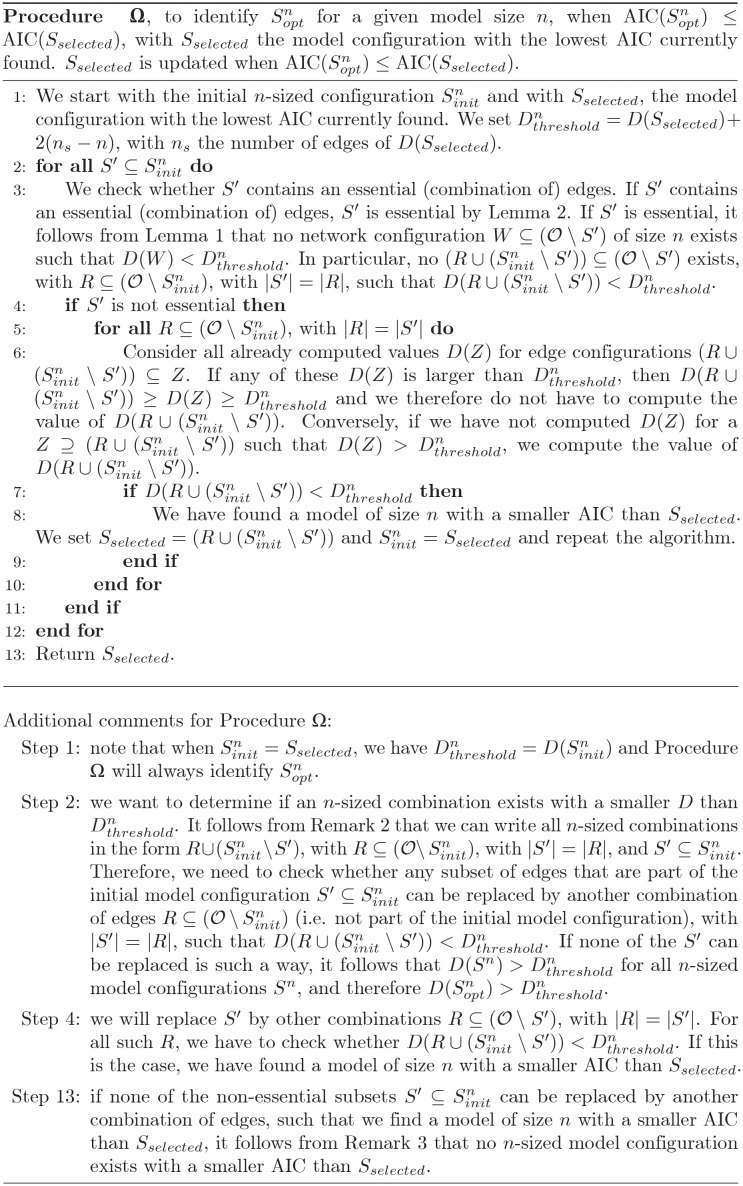



## Results

### Validation

#### Four network motifs consisting of three cytokines

**Fig 5:**
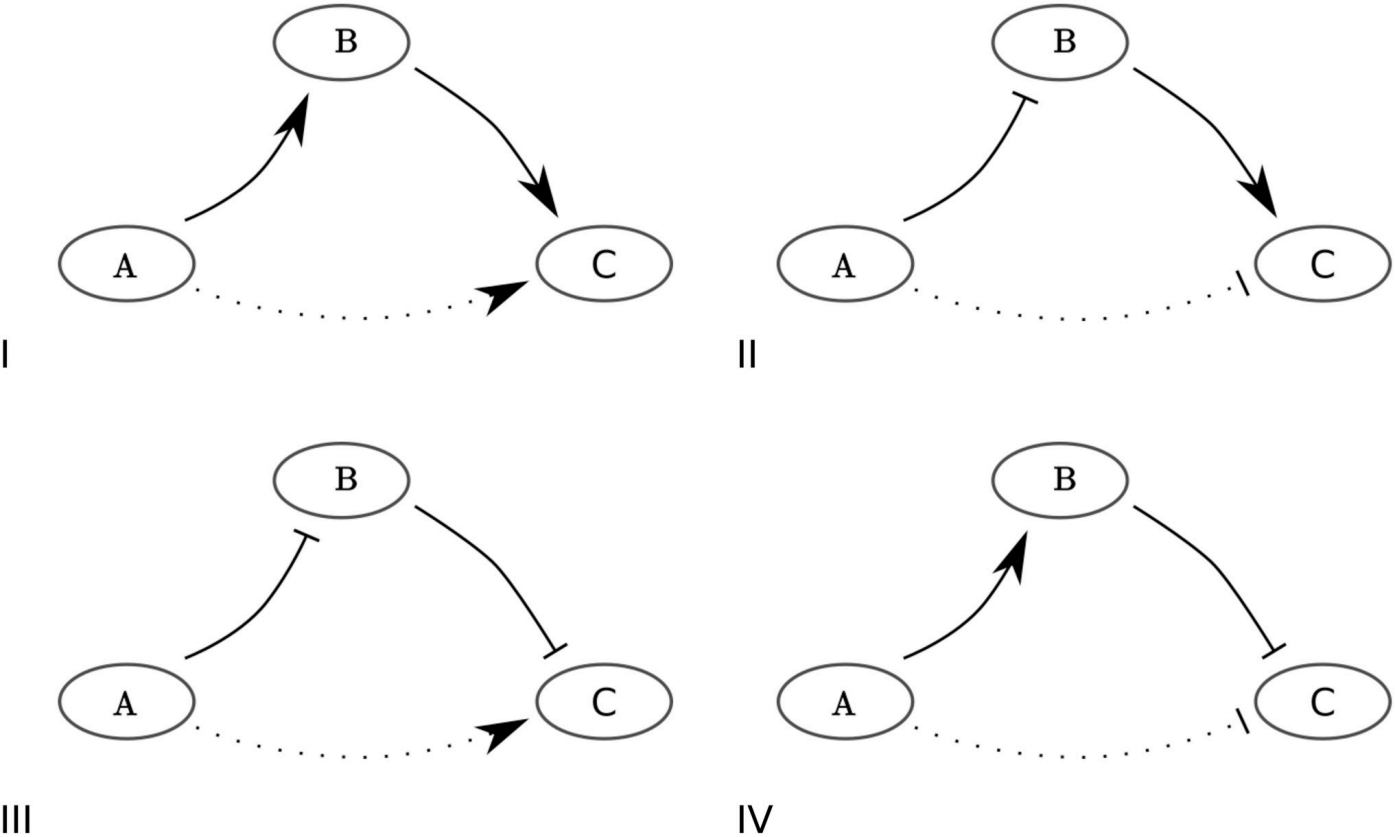
The four validation network motifs. Each motif consists of three cytokines and two directed edges. Inhibition of cytokine *A* has a negative (I and III) or positive (II and IV) effect on the secretion rate of cytokine *C*. However, this effect is indirect (via cytokine *B*), and the dotted edges between *A* and *C* do not exist.

We validate our method using synthetic data. We will first consider four network motifs consisting of three nodes and two directed edges (Fig 5). We will later explore a larger five node, ten edge network (Section “Test model consisting of five cytokines”). Each of the four motifs consists of two edges, and a third, spurious edge that might arise when a network is constructed based on statistical analysis (Fig 5, solid and dotted lines for the real and spurious edges respectively). We use our method to distinguish between real and spurious edges. For each of the four motifs, we generate synthetic datasets of cytokine secretion measurements for cytokine A, B, and C at a single point in time.

**Fig 6:**
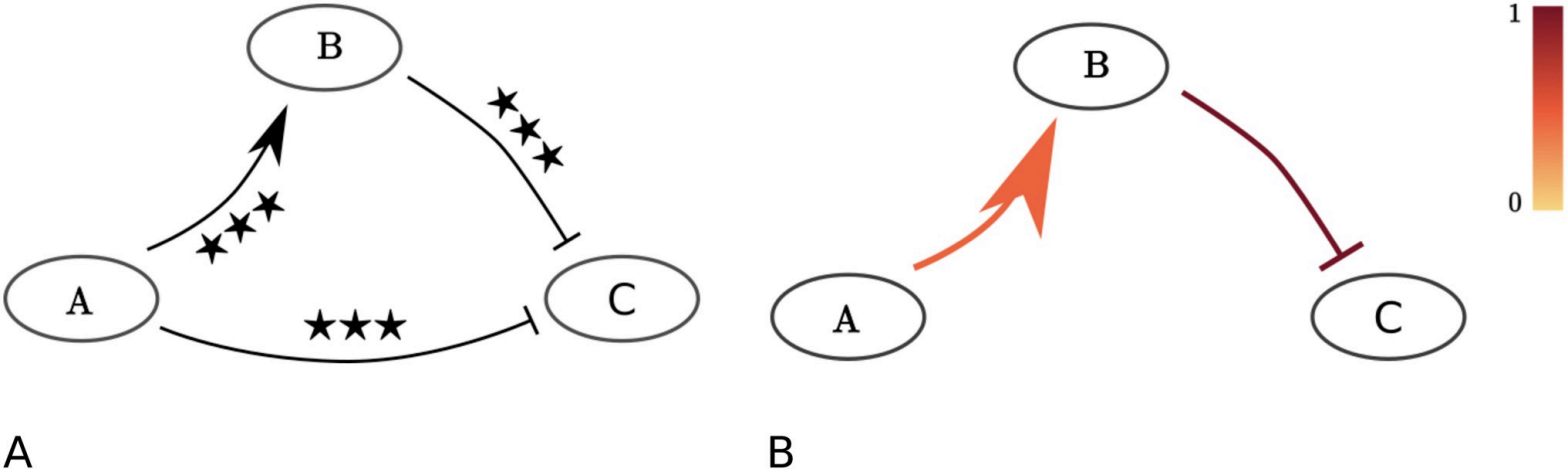
Starting with a set of statistically significant edges (left), we use the AIC to select an optimal model that balances model complexity and fit (right). A: Network extracted using statistical analysis of the synthetic data presented in Fig 1B, corresponding to the toy model shown in Figs 1A and 5IV. Two sample t-test: ^★★★^p≤ 0.001, fdr corrected. B: The two-edge network configuration selected by the AIC (see Fig 2), again based on the synthetic dataset presented in Fig 1B. Note the AIC has selected the network configuration that was used to generate the synthetic data set. Edge widths are proportional to the value of the corresponding edge parameter *α*_*u*,*i*_ and *β*_*v*,*i*_ that minimized *D* for the shown network configuration. Edges are coloured proportional to the increase in *D* when they are removed from the shown network configuration.

We generate synthetic data by simulating Eq (1). We assume our measurements are taken at *t* = 16h, and assume the stimulus *s*_*i*_ = 1 for all *i* ∈ {*A*, *B*, *C*}. We further assume we have measurements for the stimulus alone, and the stimulus in the presence of a cytokine receptor blocker for each of the three cytokines. When no up- or down-regulatory edge between two cytokines exists in our constructed network, we set the corresponding parameter *α*_*u*,*i*_ or *β*_*v*,*i*_ to zero; in particular if cytokine *p* is subject to its corresponding blocker then *α*_*p*,*i*_ = *β*_*p*,*i*_ = 0 for all *i*. Otherwise, we set *α*_*u*,*i*_ or *β*_*v*,*i*_ to 0.5. We assume we have 15 measurements for each experimental condition, for instance corresponding to measurements obtained from 15 individual donors. We add normally-distributed noise with a standard deviation of 0.25 to all log10-transformed synthetic data points. This level of noise is similar to the noise observed in the experimental IL23 dataset. We statistically analyse our synthetic data set. For each added cytokine blocker and each cytokine, we test whether the change is significant using a 2-sample t-test (*p* ≤ 0.05, fdr corrected). We will consider a network edge from cytokine *i* to cytokine *j* statistically significant, when the change in secretion of cytokine *j* after the blockade of the cytokine *i* receptor is statistically significant. The dataset presented in Fig 1B was generated in the way described, with *α*_*A*,*B*_ = *β*_*B*,*C*_ = 0.5 and all other *α*_*u*,*i*_ and *β*_*v*,*i*_ set to zero (corresponding to the network motif shown in Figs 1A and 5IV). We show the three network nodes, and the three identified significant edges in Fig 6A. Note this network includes both real edges, and a spurious edge. As we have seen before, the AIC selects the model that was used to generate this synthetic dataset, balancing model complexity and fit (Figs 2 and 6B).

We note that when we compute *D* for a network configuration, we work with a known standard deviation *σ*_*i*_, of the log-transformed data for each cytokine *i*. We determine *σ*_*i*_ from the data as follows. We compute the difference between each measurement and the mean of all measurements for that experimental condition and cytokine, where each measurement corresponds to the log10 transformed secretion rate of one cytokine, experimental condition, and donor. We compute the variance by normalizing the square of these differences by one minus from the sample size for each cytokine and compute the standard deviation as the square root of the variance.

We generated 100 synthetic datasets for each of the four motifs shown in Fig 5. For each of the 400 datasets, the two edges used to generate the data were statistically significant (*p* ≤ 0.05, fdr corrected). In 100%, 99%, 99%, and 100% of cases, the third, spurious edge was statistically significant as well for each of the four network motifs respectively (*p* ≤ 0.05, fdr corrected). Further, in 32 of the 400 cases, one or more of the remaining fifteen edges (not including the three edges considered) was found to be statistically significant (*p* ≤ 0.05, fdr corrected). When we increase the value of the two ‘true’ edge parameters *α*_*u*,*i*_ and/or *β*_*v*,*i*_ from 0.5 to 0.75, in all 400 cases the third, spurious edge was found to be significant (*p* ≤ 0.05, fdr corrected). Conversely, when we decrease the value of the ‘true’ edge parameters from 0.5 to 0.1, both ‘true’ edges are significant in only 93%, 70%, 59% and 93% of cases. In 28%, 14%, 13% and 29% of the 400 cases a spurious third edge was found to be significant in this case (*p* ≤ 0.05, fdr corrected). All spurious results are anticipated to be small number effects, though such numbers of samplings reflect current experimental protocols. In the following, we will only proceed to consider the case where the value of *α*_*u*,*i*_ and/or *β*_*v*,*i*_ is set to the intermediate value of 0.5 for edges that are present. This is motivated in particular by the context of the experimental study whose datasets we use, namely the identifcation of prospective cytokine targets and thus cytokines that have at least an intermediate impact on cytokine regulation, rather than a weak impact.

For each of the four motifs, we estimate the model fit to each of the synthetic datasets (100 datasets per motif). We compute the model fit for the network extracted by statistical analysis and all sub-networks (by minimizing the log likelihood, with D=-2log(L) and the likelihood L defined in Eq (3)). We then apply the AIC for model selection. In 86%, 84%, 83%, and 88% of cases for each of the four motifs did the AIC select the ‘true’, 2-edge model and in 8%, 9%, 12%, and 8% of cases a model including the spurious edge from Cytokine A to Cytokine C, i.e. A → C (test motifs I and III) or A ⊣ C (test motifs II and IV). We recall that in 32 of the 400 cases, spurious edges, other than the one from Cytokine A to Cytokine C were found to be significant. For these datasets, all sub-configurations including such significant edges were considered. Of these, in 25 cases, a model was selected by the AIC including one or more of such spurious edges. The weighting of the spurious edges included in selected model configurations was small (average edge weight < 0.05) compared to an average of 0.5 for the ‘real’ edges, highlighting that spurious edges are still subdominant in the predicted interactions. We did not observe any false negatives, i.e. both ‘true’ edges used to generate the synthetic data were selected in all 400 cases.

#### Test model consisting of five cytokines

**Fig 7:**
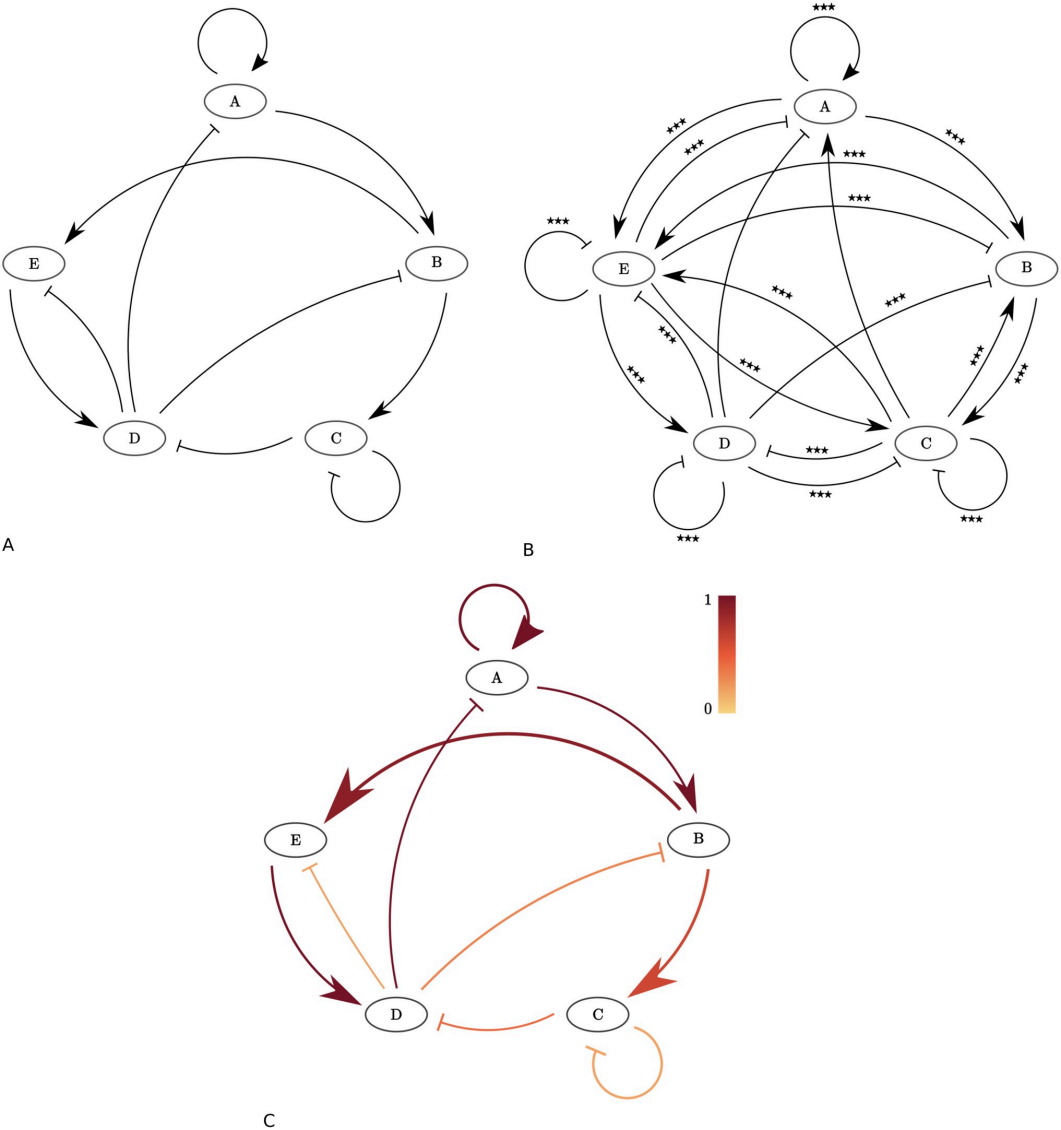
The validation test model consisting of five cytokines and ten edges. A: The test model. To construct this model configuration, we included the four network motifs we considered before (eight edges, Fig 5), and added two self-edges. B: Network extracted using statistical analysis from the example synthetic data set (Section “Validation”). Two sample t-test: ^★★★^p≤ 0.001, fdr corrected. Ten of the shown edges are part of the test model and were used to generate the synthetic dataset. The other edges are spurious. C: The ten-edge network configuration selected by the AIC, applied to our example synthetic dataset. Edge widths are proportional to the value of the corresponding edge parameter *α*_*u*,*i*_ and *β*_*v*,*i*_ that minimized *D* for the shown network configuration. Edges are coloured proportional to the increase in *D* when they are removed from the shown network configuration.

We repeated the procedure for a more extended test network (Fig 7A). We consider five cytokines: *A*, *B*, *C*, *D*, and *E*. We constructed the network such that all four previously explored motifs are present in the larger network. We also included a positive and negative self-edge. We again generated synthetic data by simulating Eq (1). We assume we have 15 measurements for each experimental condition at *t* = 16h and added noise with a standard deviation of 0.25 to the log-transformed synthetic data set. We again assume *s*_*i*_ = 1 for all cytokines *i* ∈ {*A*, *B*, *C*, *D*, *E*} and *α*_*u*,*i*_ = *β*_*v*,*i*_ = 0.5 for all existing edges, with zero values for non-existing edges including those removed by a cytokine blocker, as previously described for the three cytokine synthetic model. We confirmed using one hundred synthetic datasets that all ‘real’ edges are always statistically significant for these values of *α*_*u*,*i*_ and *β*_*v*,*i*_. On average, 11.4 additional, spurious edges were found to be statistically significant. We show the network extracted by statistical analysis for one of these datasets in Fig 7B. In addition to the ten ‘true’ edges, ten additional edges were detected in this case (Fig 7B). The AIC selects the ‘true’ model, i.e. the model used to generate the synthetic dataset, as the optimal model in this case, removing all ten spurious edges (Fig 7C). In total, we computed the *D* for 120 network configurations to select this network, approximately 0.01% of ∑k=120(20k)=220-1>106, the total number of possible network configurations using 20 different edges. Because of the computational requirements of running the selection algorithm (approximately 6.1h per synthetic dataset on a Macbook Pro laptop with a 2.3 GHz Intel Core i5 processor), we did not apply our method to all one hundred synthetic datasets. For each of the one hundred synthetic datasets, we compared the AIC of the true ten edge model with the AIC of all eleven edge models constructed by adding one of the identified spurious edges to the set of true edges at a time. In 53 cases, the addition of none of the spurious edges (11.4 per dataset on average) did improve the AIC of the true 10 edge model. The weighting of the spurious edges in the 53 configurations that improved the AIC in comparison to the AIC of the true ten edge model was small (average edge weight < 0.003). The average of the ten ‘real’ edges was 0.5 for these configurations. The addition of one of the spurious edges did not improve the model fit in 1048 out of 1137 edges. Hence, in 92.2% of cases, the addition of a false negative edge identified by the statistical analysis to the true model configuration did not improve the AIC.

#### Parameter identifiability

**Fig 8:**
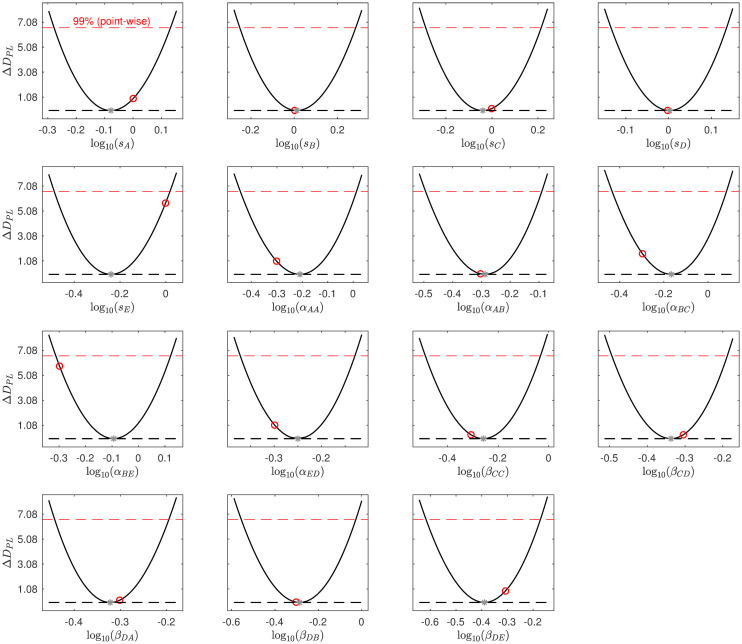
Profile likelihood plots for the ten edge parameters of the selected 10-edge 5 cytokine test model. The parameter *θ*_*m*_ ∈ ***θ*** was varied over a broad range of values and for each fixed value of *θ*_*m*_, the increase in $D_{PL}\left(\theta_{m}\right)=\min_{{\tilde{\theta }}_{m}}-2\;\text{log}(L(\theta ))$ was computed, with L(θ) the likelihood function as defined in Eq (3), and ${\tilde{\theta }}_{m}=\left\{\theta_{1},\ldots ,\theta_{m-1},\theta_{m+1},\theta_{N}\right\}$. The 99% confidence interval threshold is shown as a red dashed line. The parameter values used to generate the synthetic dataset are shown as red dots. The parameter values resulting in the minimal $D=\min_{\theta }-2\;\text{log}(L(\theta ))$ are shown as grey stars. This figure has been generated using the Matlab environment ‘Data2Dynamics’ [32, 33].

**Fig 9:**
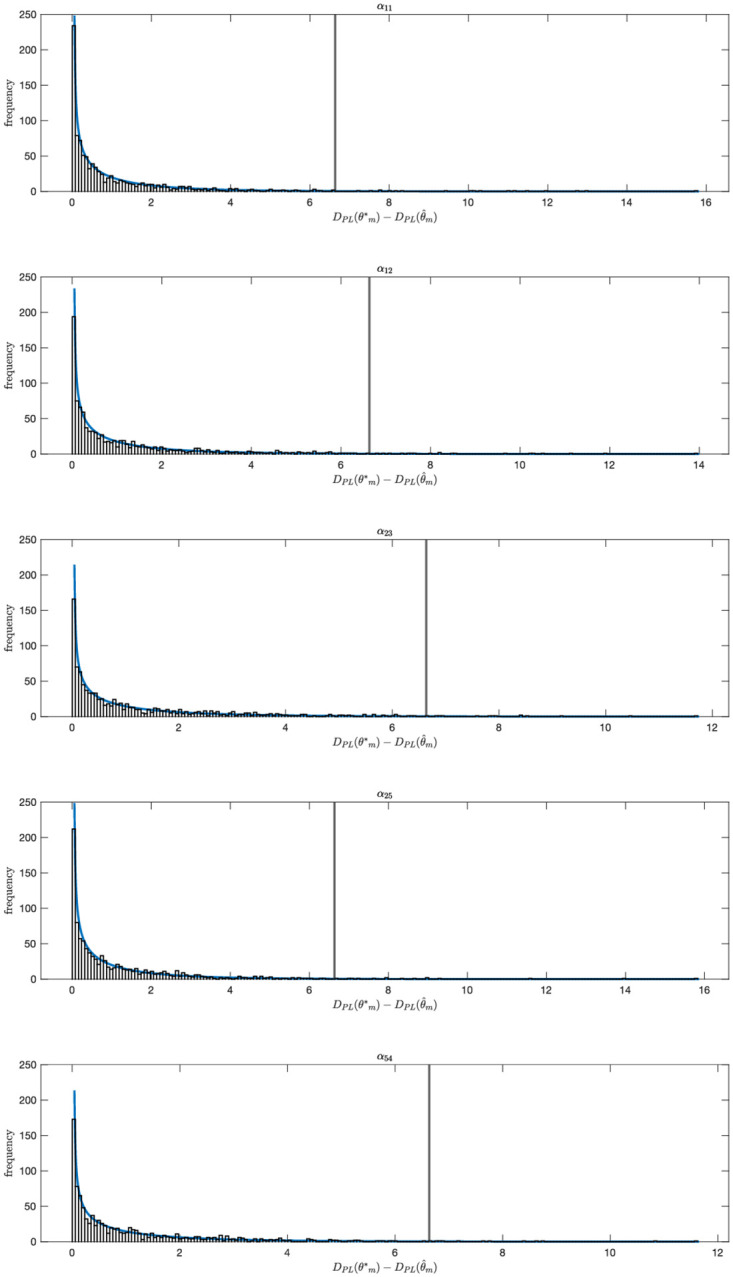
Validation of the coverage of the confidence intervals. The *χ*^2^ distribution with one degree of freedom (blue) and, for a 1000 independent datasets, and for five edge parameters *θ*_*m*_ ∈ ***θ*** of the 10-edge 5 cytokine test model, a histogram of the values of $D_{PL}\left(\theta_{m}^{*}\right)-D_{PL}\left({\hat{\theta }}_{m}\right)$, with $D_{PL}\left(\theta_{m}\right)=\min_{{\tilde{\theta }}_{m}}-2\;\text{log}(L(\theta ))$, ${\tilde{\theta }}_{m}=\left\{\theta_{1},\ldots ,\theta_{m-1},\theta_{m+1},\ldots ,\theta_{N}\right\}$. Here, $\theta_{m}^{*}=0.5$ is the true value of *θ*_*m*_ and $\hat{\theta }$ its estimated value by log-likelihood minimization. In 99% of all cases, the value of $D_{PL}\left(\theta_{m}^{*}\right)-D_{PL}\left({\hat{\theta }}_{m}\right)$ is smaller than *χ*^2^(0.99, 1) (vertical grey line).

**Fig 10:**
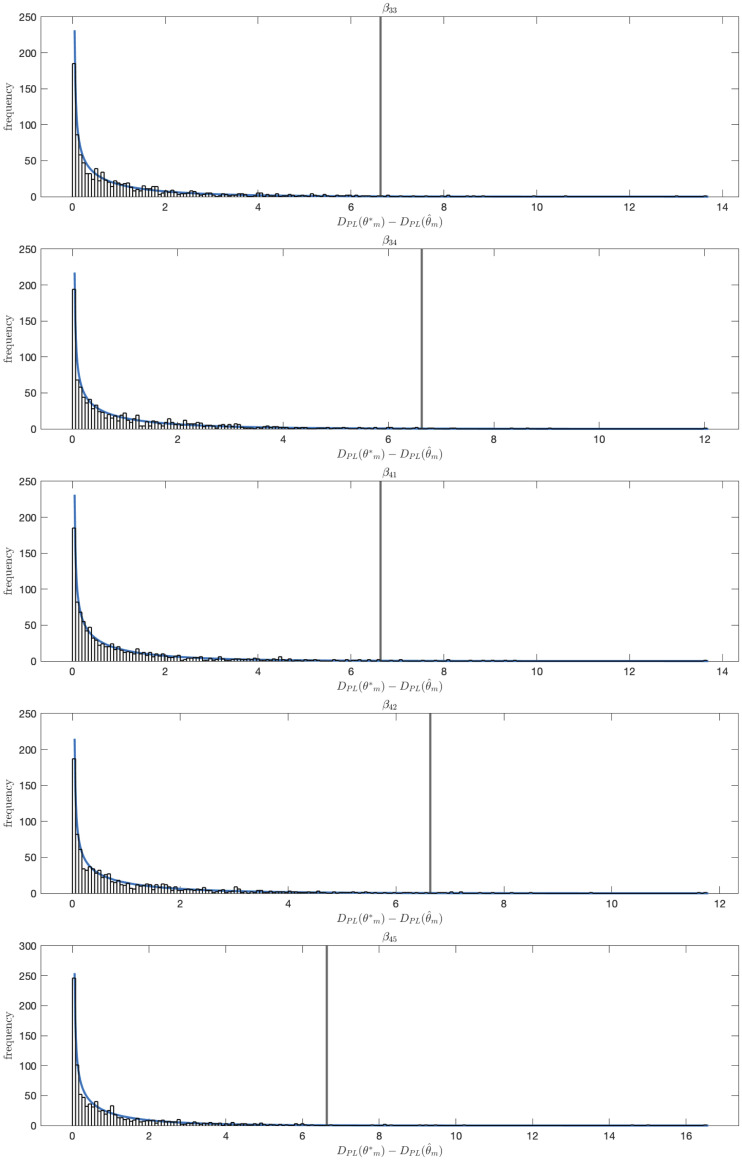
Validation of the coverage of the confidence intervals. The *χ*^2^ distribution with one degree of freedom (blue) and, for a 1000 independent datasets, and for the remaining five edge parameters *θ*_*m*_ ∈ ***θ*** of the 10-edge 5 cytokine test model, a histogram of the values of $D_{PL}\left(\theta_{m}^{*}\right)-D_{PL}\left({\hat{\theta }}_{m}\right)$, with $D_{PL}\left(\theta_{m}\right)=\min_{{\tilde{\theta }}_{m}}-2\;\text{log}(L(\theta ))$, ${\tilde{\theta }}_{m}=\left\{\theta_{1},\ldots ,\theta_{m-1},\theta_{m+1},\ldots ,\theta_{N}\right\}$. Here, $\theta_{m}^{*}=0.5$ is the true value of *θ*_*m*_ and $\hat{\theta }$ its estimated value by log-likelihood minimization. In 99% of all cases, the value of $D_{PL}\left(\theta_{m}^{*}\right)-D_{PL}\left({\hat{\theta }}_{m}\right)$ is smaller than *χ*^2^(0.99, 1) (vertical grey line).

We consider local identifiability of the edge parameters using the Profile Likelihood Approach [32] (Section “S2 Appendix”). To compute the profile likelihood based confidence intervals we varied the value of each model parameter *θ*_*m*_ ∈ ***θ*** over a broad range of values and for each value computed the increase in $D_{PL}\left(\theta_{m}\right)=\min_{{\tilde{\theta }}_{m}}-2\;\text{log}(L(\theta ))$, with L(θ) the likelihood function as defined in Eq (3), and ${\tilde{\theta }}_{m}=\left\{\theta_{1},\ldots ,\theta_{m-1},\theta_{m+1},\ldots ,\theta_{N}\right\}$. Hence for each fixed value of *θ*_*m*_ we find the minimal *D*_*PL*_(*θ*_*m*_) by fitting all other parameters to the data. This allows us to compute likelihood based parameter value confidence intervals for each parameter with threshold value *ζ*, the *ζ* quantile of the $\chi_{1}^{2}$ distribution. The true value of *θ*_*m*_ will lie within the *ζ* level confidence interval with probability *ζ* [32]. Infinite confidence intervals in the parameter value space correspond to parameter non-identifiability. We calculated 99% parameter confidence intervals for each of the edge parameters for the selected 10-edge model shown in Fig 7C (Fig 8). We recall that the log10-transformed values of the ten edge parameters used to generate the synthetic data are log_10_0.5 = −0.301. These values are contained in the 99% confidence intervals of all ten original edge parameters (Fig 8). We validated the coverage of the confidence intervals by calculating $D_{PL}\left(\theta_{m}^{*}\right)-D_{PL}\left({\hat{\theta }}_{m}\right)$, for every edge parameter *θ*_*m*_ for 1000 independent datasets, with $\theta_{m}^{*}$ the true value of the parameter and ${\hat{\theta }}_{m}$ its estimated value. We show the histograms of $D_{PL}\left(\theta_{m}^{*}\right)-D_{PL}\left({\hat{\theta }}_{m}\right)$ and confirm they approximate a *χ*^2^(*ζ*, 1) distribution (Figs 9 and 10). We note that $D_{PL}\left({\hat{\theta }}_{m}\right)=-2\;\text{log}\left(L\left(\hat{\theta }\right)\right)$.

#### Model predictions

**Fig 11:**
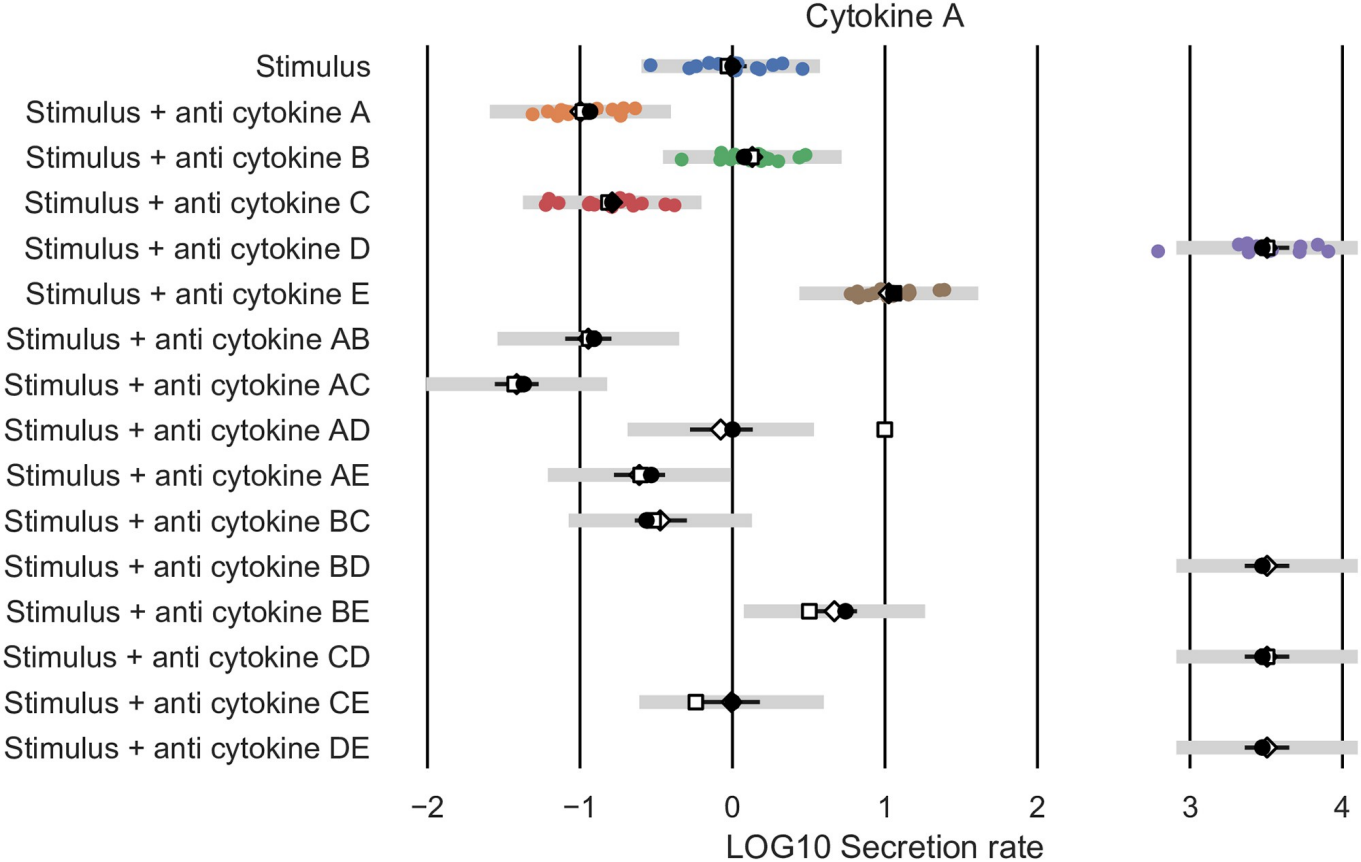
The synthetic dataset for Cytokine A (coloured dots). Model simulations for the mean cytokine secretion of Cytokine A with the edge weights set to the values used to generate the example synthetic dataset (black dots) and model simulations with the edge weights fitted to the synthetic dataset, both for the model configuration containing all significant edges (white, squares), and the selected ten edge model (white, diamonds). The Log10 transformed values 12.4 and 69.5, corresponding to the ‘Anti cytokine BD’ and ‘Anti cytokine DE’ simulations of the model configuration containing all significant edges are not shown for visibility reasons. In 99% of cases, we expect an experimental datapoint (grey lines, validation profile likelihood confidence intervals), or the true mean cytokine secretion rate (black lines, prediction profile likelihood confidence intervals) to lie within the shown intervals for our ten edge test model predictions.

**Fig 12:**
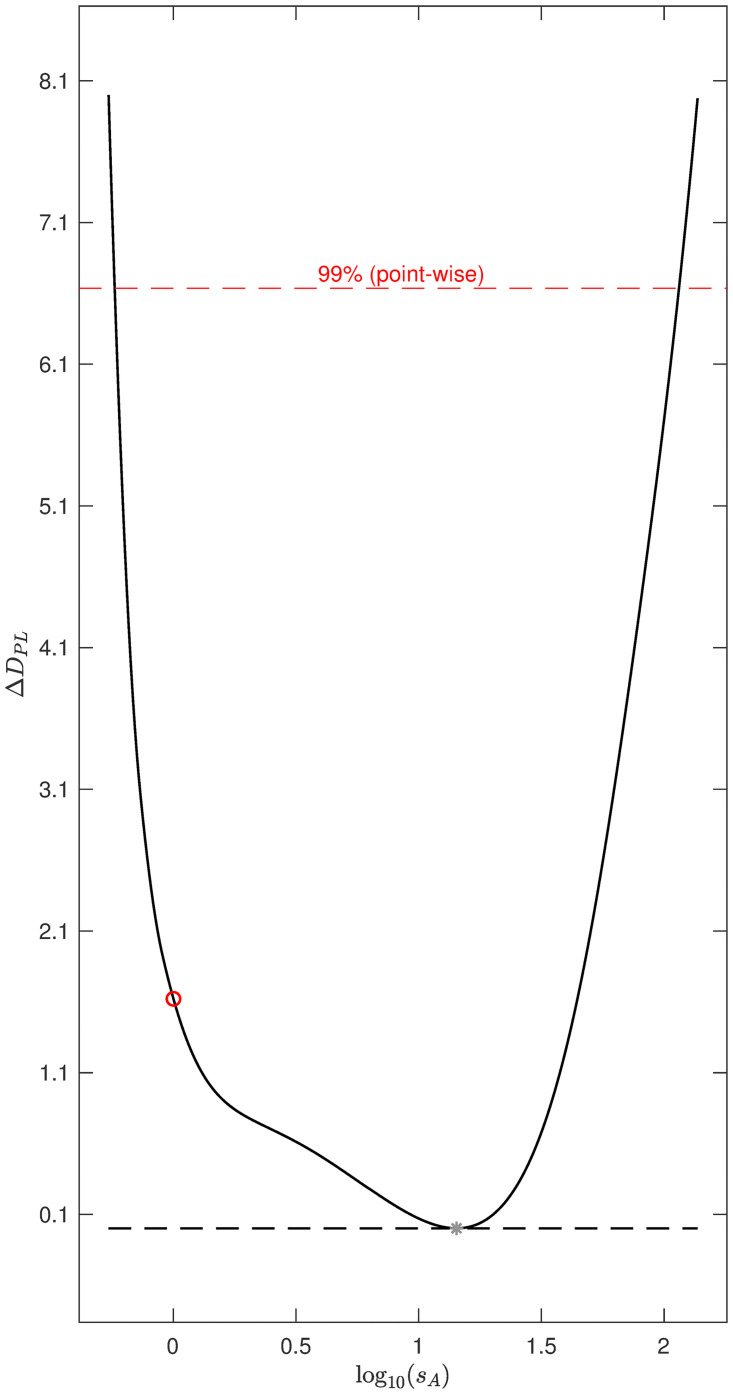
Profile likelihood plot for *s*_*A*_ for the 20-edge 5 cytokine test model. The parameter *θ*_*m*_ = *s*_*A*_ ∈ ***θ*** was varied over a broad range of values and for each fixed value of *θ*_*m*_, the increase in $D_{PL}\left(\theta_{m}\right)=\min_{{\tilde{\theta }}_{m}}-2\;\text{log}(L(\theta ))$ was computed, with L(θ) the likelihood function as defined in Eq (3), and ${\tilde{\theta }}_{m}=\left\{\theta_{1},\ldots ,\theta_{m-1},\theta_{m+1},\theta_{N}\right\}$. The 99% confidence interval threshold is shown as a red dashed line and corresponds to a two order of magnitude interval for *s*_*A*_. The parameter value used to generate the synthetic dataset is shown as a red dot. The parameter value resulting in the minimal $D=\min_{\theta }-2\;\text{log}(L(\theta ))$ is shown as a grey star. This figure has been generated using the Matlab environment ‘Data2Dynamics’ [32, 33].

Using the shown model selected by the AIC (Fig 7C), we can generate predictions of (untested) combinations of cytokine receptor blockades. We show predictions for the mean Cytokine A secretion rate by the model as selected by AIC (white diamonds), predictions using the *N* = 20-edge model containing all statistically significant edges (white squares), and the ‘true’ mean values obtained by setting *s*_*i*_ = 1 and *α*_*u*,*i*_ = *β*_*v*,*i*_ = 0.5 for all ten existing edges (black dots, Fig 11). For the 10-edge and 20-edge model simulations, we have fitted the 10- and 20-edge parameter values respectively to the synthetic dataset (coloured dots). We note that the predictions by the selected 10-edge model match the ‘true’ values closely. The larger, 20-edge model does not perform well. We note that the secretion rate of Cytokine A in the presence of a receptor blocker of both cytokine A and D, both B and D, and of both D and E is overestimated by the 20-edge model simulations. Although the 20-edge model more closely fits the data than the 10-edge model, the predicted cytokine secretion rate of Cytokine A in the presence of a receptor blocker of both cytokine D and E is 10^70^ times higher than under control conditions. This clearly is a biologically implausible prediction, resulting from a very large estimated value of the parameter *s*_*A*_ = 10^1.2^ = 14 for the 20-edge model. We note that the 99% profile likelihood confidence interval for parameter *s*_*A*_ for the 20-edge model ranges from 10^−0.2^ = 0.6 to 10^2.1^ = 115, while the true value, used to generate the synthetic data set is *s*_*A*_ = 10^0^ = 1 (Fig 12). In contrast, the 99% profile likelihood confidence interval for parameter *s*_*A*_ for the 10-edge model ranges from 10^−0.3^ = 0.5 to 10^0.1^ = 1.4 (Fig 8). Using the selected, 10-edge model reduces overfitting of the data and results in more accurate model predictions.

To obtain confidence intervals for our model predictions, we follow the Prediction Profile likelihood approach by Kreutz et al. [34]. In a way analogous to the Profile likelihood approach, we define for each model simulation *y*_*i*,*j*_ = *z*, for cytokine *i* and experimental condition *j*: $D_{PPL}\left(z\right)=\min_{\bar{\theta }}-2\;\text{log}(L(\theta ))$, where the parameter values $\bar{\theta }$ are constrained such that the model simulation *y*_*i*,*j*_ takes on the value *z*. Hence for each fixed value of *y*_*i*,*j*_ = *z* we find the minimal *D*_*PPL*_(*z*) by fitting all parameters to the data, subject to the constraint *y*_*i*,*j*_ = *z*. This allows us to compute likelihood based prediction confidence intervals for each model simulation *y*_*i*,*j*_ with threshold value *ζ*, the *ζ* quantile of the $\chi_{1}^{2}$ distribution. Our model simulations *y*_*i*,*j*_ are estimates of the mean cytokine secretion rate. The true mean cytokine secretion rate will lie within the *ζ* level confidence interval with probability *ζ*. We calculated 99% confidence intervals for each of the model simulations of the selected 10-edge model, using the Prediction profile likelihood approach (Section “S2 Appendix”). We confirm that all true mean cytokine secretion rates (black dots) are contained in the 99% confidence intervals for our ten edge test model predictions (black lines, Fig 11). We also calculated 99% Validation confidence intervals for each of the model simulations of the selected 10-edge model, using the Validation profile likelihood approach (Section “S2 Appendix”). In 99% of cases, we expect an experimental datapoint to lie within the Validation profile confidence intervals of the model simulations. We observe that only one data-point (left purple dot) is not contained in the 99% Validation confidence intervals of the 10-edge model simulations.

#### Application to a dataset on IL23 regulation

We apply our method to an experimental dataset of stimulated peripheral blood mononuclear cells (PBMCs) to study the regulation of the cytokine IL23, a key cytokine in IBD pathogenesis. The application of our method to an extended version of this dataset was published before [35]. In contrast to the study presented in [35], here we will validate several of our model predictions against experimental data that was not used for model fitting to demonstrate the predictive capability of this modelling framework. The dataset we used for model fitting contains data on the secretion of six relevant cytokines (TNF*α*, IL1*α*, IL1*β*, IL6, IL10, and IL23) by monocytes (live CD14+ cells) at *t* = 16h after stimulation with the bacterial cell-wall component LPS (*n* = 17 healthy donors). Cytokine secretion was measured by flow cytometry in the presence of LPS and aTNF, aIL6R, aIL10R, aIL1*α*, aIL1*β* and aIL1R, where aIL1R blocks both the IL1*α* and IL1*β* receptor, a ≡ anti, and R ≡ receptor.

Further, the effects of the exogenous addition of LPS and the cytokine IFN*γ* were measured and, in the model network developed below from monocyte cytokine secretion measurements, the impact of IFN*γ* is treated analogously to the LPS stimulus, as we briefly motivate. In particular, IFN*γ* is a cytokine that is only produced by a distinct subset of immune cells, for instance memory CD4^+^ *αβ*T cells (in particular Th1 and Th1/17 subsets), with a well-established role in promoting IL-12 and IL-23 synthesis in myeloid lineage cells, including monocytes. The latter express IFNGR1 and IFNGR2 (Interferon gamma receptors 1,2) *but do not produce IFNγ themselves* [36–38]. This is further evidenced by the RNA-sequencing-based analysis of sorted immune cell subsets from peripheral blood mononuclear cells (which include monocytes), such as the Monaco dataset [39], demonstrating expression of the receptors IFNGR1 and IFNGR2 but not IFN*γ*. In addition, the experimental study specifically aimed to analyse the cytokine network involved in monocyte IL-23 synthesis, including stimulation with recombinant human IFN*γ* as a benchmark IL-23 synthesis promoting factor for the set of stimulation conditions of interest [35]. Our analysis confirmed that recombinant human IFN*γ* does indeed upregulate monocyte IL-23 synthesis when added to LPS-stimulated and LPS+aIL-10R stimulated cultures and that blocking IFN*γ* signalling in the context of LPS and LPS+aIL-10R stimulation does not affect IL-23 synthesis [35]. In contrast, IL-1 signalling is a prerequisite for monocyte IL-23 production as evidenced by the fact blocking the receptor IL-1R1 does impair IL-23 production in both LPS and LPS+aIL-10R stimulations [35]. These latter data further suggest that the level of IFN*γ* produced by LPS and LPS+aIL-10R in the short ex-vivo stimulation of the experiment is not sufficient to drive IL-23 expression [35]. Hence while exogenous IFN*γ* is introduced in amounts that can be influential, there is substantial evidence to support the modelling assumption that the cells in the experiment do not produce IFN*γ* even though they are influenced by exogenous IFN*γ*.

To construct suitable datasets we first multiplied the percentage of monocytes expressing a cytokine with the mean fluorescence intensity (MFI) of the monocytes expressing that cytokine to obtain a measure (PMFI, arbitrary units) of the amount of cytokine present within the monocytes. We assume each produced cytokine is secreted after a fixed, small amount of time and our measure of cytokines present within the monocytes (PMFI) therefore correlates with the cytokine secretion rate. For a large proportion of the studied experimental conditions, the logarithm of the PMFI data significantly deviated from a normal distribution (false discovery rate (fdr) corrected Lilliefors test for normality, p<0.05). We therefore attempted to remove the lack of log-normality by rescaling the data. Ideally, we would have liked to rescale by dividing each sample by the sum of all studied experimental conditions, so as not bias any one experimental condition in the rescaling. However, as not every experimental condition was measured for each donor, we chose to rescale by the sum of two completely measured experimental conditions (i.e. conditions that were measured for each donor). In particular, we chose the ‘LPS’ and ‘LPS + aIL10R’ conditions for rescaling as they were both measured for all 17 donors. To clarify, for each donor, we rescaled the sample PMFI value for each experimental condition by dividing by the sum of the sample PMFI values of the ‘LPS’ and ‘LPS + aIL10R’ conditions for that particular donor. In particular the use of two datasets is motivated by the fact that if only dataset was used, say ‘LPS’, the rescaled LPS data would be unity with zero standard deviation and would have to excluded from the likelihood, Eq (3).

After this rescaling the residuals (i.e. the difference from the group mean) of the ‘LPS’ samples, the residuals of the ‘LPS + aIL10R’ samples, and the residuals of all other conditions combined did indeed not significantly deviate from a log-normal distribution (fdr corrected Lilliefors test for normality on the log of the rescaled data, p<0.05). We determined the fixed cytokine specific standard deviation *σ*_*i*_ for each cytokine *i* as before, but without the datapoints corresponding to the ‘LPS’ and ‘LPS + aIL10R’ conditions that were used for rescaling. We determined two separate, experimental condition and cytokine specific, standard deviations *σ*_LPS,*i*_ and *σ*_aIL10R,*i*_ of the log transformed data for the ‘LPS’ and ‘LPS + aIL10R’ samples that were used for the rescaling.

**Fig 13:**
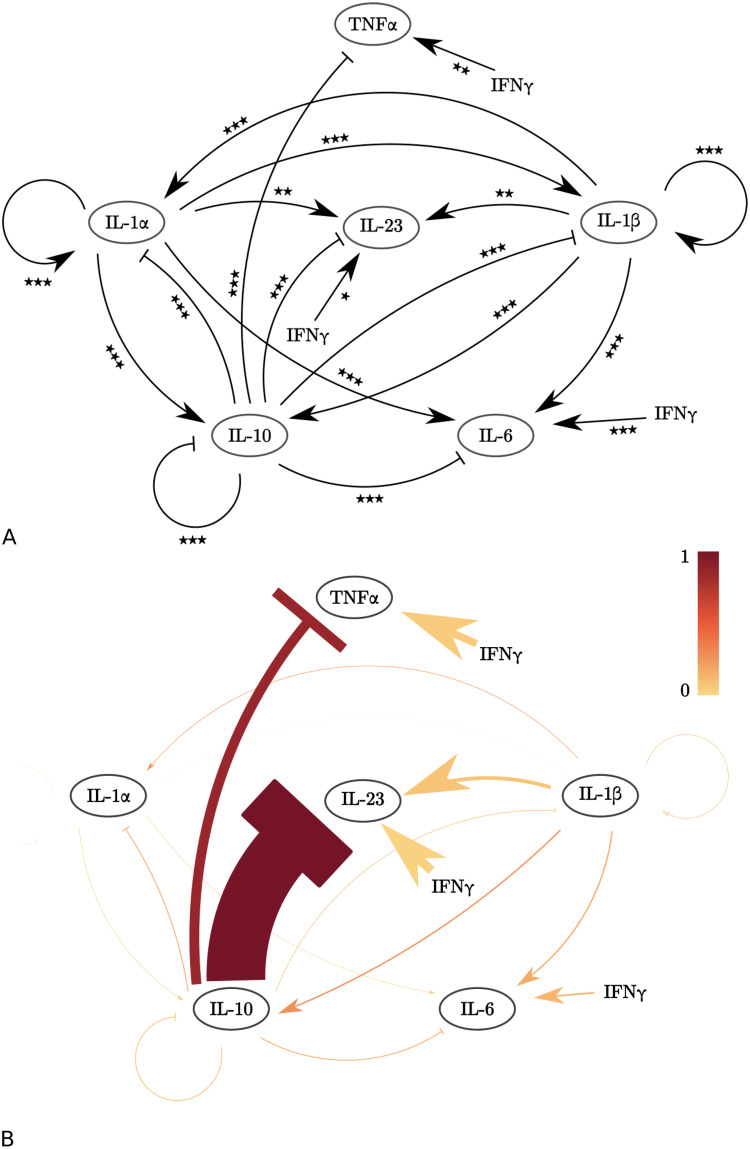
The IL23 model. A: Network extracted from the flow cytometry data set using statistical analysis (Section “Application to a dataset on IL23 regulation”) [35]. Using a two sample t-test, we test the hypothesis that the presence of inhibitor induces a change relative to the control: ^★^p≤ 0.05, ^★★^p≤ 0.01, ^★★★^p≤ 0.001, fdr corrected. B: The eighteen-edge network configuration selected by the AIC. Edge widths are proportional to the value of the corresponding edge parameter *α*_*u*,*i*_ and *β*_*v*,*i*_ that minimized *D* for the shown network configuration. Edges are coloured proportional to the increase in *D* when they are removed from the shown network configuration.

**Fig 14:**
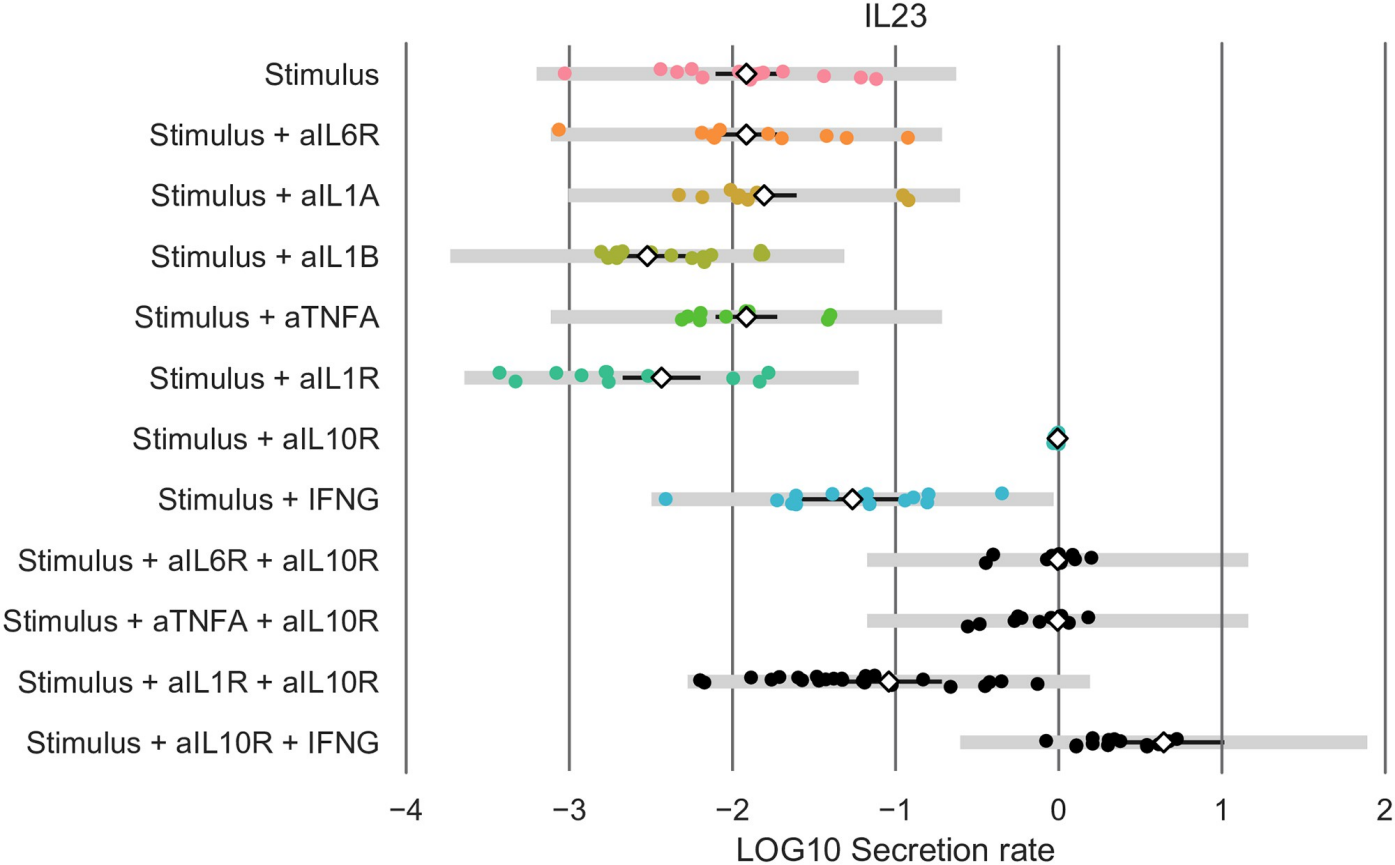
Model predictions by the selected, eighteen edge model for IL23 (white, diamonds) for various experimental conditions. We show the cytokine secretion dataset used for fitting (coloured dots, top eight rows) and the validation dataset (black dots, bottom four rows). We note that cytokine secretion data for all six cytokines (TNF*α*, IL1*α*, IL1*β*, IL6, IL10, IL23) was used for fitting, but only the IL23 data is shown in this figure. In 99% of cases, we expect an experimental data-point (grey lines, validation profile likelihood confidence intervals), or the true mean cytokine secretion rate (black lines, prediction profile likelihood confidence intervals) to lie within the shown intervals for our selected eighteen edge model predictions.

We applied our method to the rescaled dataset. In the log-likelihood function (Eq (3)), we replaced *σ*_*i*_ by *σ*_LPS,*i*_ or *σ*_aIL10R,*i*_ for data points associated with the experimental conditions ‘LPS’ and ‘LPS+aIL10R’, respectively. We implemented the addition of IFN*γ* by multiplying the cytokine secretion rate *y*_*i*_(*t*) (Eq (1)) with an additional term (1+ *α*_IFN*γ*,*i*_), where the value of *α*_IFN*γ*,*i*_ corresponds to the edge width of the effect of IFN*γ* on cytokine *i* secretion. We show the set of statistically significant edges (Fig 13A), and selected model (Fig 13B), consisting of 18 edges. We observe that IL23 is directly regulated by IL10, IL1*β* and IFN*γ*, but not by TNF*α*. Using the selected model, we predicted the effects of pairwise inhibition of IL10 and TNF, IL1*α*/*β*, and IL6, and the effects of inhibiting IL10 and adding IFN*γ*. These model predictions were experimentally validated. In particular all predictions, white diamonds in Fig 14 in the lower four rows of data presented in Fig 14, are close to the mean of the experimental data and well within the 99% confidence intervals of the data (black lines).

## Discussion

We present a novel method to analyse the effects of the blockage of single cytokines on the secretion rate of other cytokines of interest. We use ordinary differential equation (ODE) based models and the Akaike information criterion (AIC) to identify a cytokine interaction network and associated edge weights that best fits the available data. Our method depends on the global minimization of a log-likelihood function and a deterministic trust-region approach was used combined with a multi-start strategy, as implemented in the Matlab-based modelling environment ‘Data2Dynamics’ [33].

We validated our method using synthetic datasets. We first considered four test motifs, each consisting of three cytokines and two edges (Fig 5). We observed that in 15% of the 400 synthetic datasets we studied, the models selected by the AIC contained one or more edges that were not part of the network configuration used to generate the synthetic datasets, though always with very low relative edge weights. The edges used to generate the synthetic datasets were always part of the selected models. We conclude that our method tends to select network configurations that are equal or slightly larger than the ‘real’ network configurations with only very weakly interacting additional edges. We also explored a larger five cytokine test model for model validation. The AIC selected all ten edges used to generate the synthetic dataset. The five cytokine test model predictions generated by the model configuration selected by the AIC were more accurate than the model predictions using the model configuration containing all twenty statistically significant edges. This shows the value of using the AIC in reducing over-fitting of the data, instead of considering all statistically significant edges. We confirmed all parameters were locally identifiable using a profile likelihood approach.

The algorithm Ω introduced to identify optimal subsets from the set of statistically significant edges reduces the number of network configurations for which the distance to the data *D* has to be computed while performing this task. For the five cytokine test model containing 20 edges, we only had to compute the *D* for approximately 0.01% of ∑k=120(20k)=220-1>106, the total number of possible network configurations. We note that this percentage is dependent on the number and relative strength of the various interactions, and in particular on the number of essential edges. These are edges that are part of a specific model configuration of interest which impact the model fit, such that a model configuration of any size without the essential edge fits the data worse than the original model configuration of interest. A large number of (configurations of) essential edges will greatly reduce the number of configurations for which *D* needs to be determined. The exact dependence of the efficiency of the algorithm on the relative strength of the various interactions could be explored in future work. The described algorithm calculates an optimal subset from a given set of feasible possibilities. We note that the algorithm is not dependent on a network structure and could have more general applications beyond the identification of cytokine interactions.

We applied our method to an experimental dataset to study the regulation of IL23 in the context of inflammatory bowel disease. The IL23 driven Th17 pathway has been identified as an important therapeutic target, but is still poorly understood at a mechanistic level [35, 40]. A specific application of our method to an extended version of this dataset was published before in Aschenbrenner and Quaranta et al. [35]. In line with the previously published results, we observe that the selected network indicates that in this inflammatory context, IL23 is driven by IL1, and to a lesser extent by IFN*γ*, but not by TNF*α* (Figs 13 and 14). This supports the view of IL1 and IL23 as a promising target for those patients that fail to respond to TNF*α* inhibition, the standard of care for many severe inflammatory mediated diseases, including inflammatory bowel disease (IBD). Targeting IL23, primarily via its subunit p_40_, is increasingly recognized to be an effective treatment of IBD, including for patients with anti-TNF resistance [1, 4]. Further, IL-1 receptor blockade might be effective in certain sub-types of IBD [3]. IL-1 receptor antagonist has been shown to ameliorate colitis in a Mendelian type of IBD, driven by mutations in the mevalonate kinase pathway [41]. Another regulator of interest is IL10. As the experimental data indicates that IL10 downregulates both IL23 and IL1*β*, and IL1*β* in turn upregulates IL23, one might hypothesize that the observed downregulatory effect of IL10 on IL23 is IL1*β* dependent: i.e. IL10 might not have a direct effect on IL23. However, the network predictions clearly indicate that the downregulation by IL10 of IL23 is largely IL1 independent (compare ‘Stimulus + aIL1R’ and ‘Stimulus + aIL1R + aIL10R’ in Fig 14). We note that the observed direct downregulation by IL10 of IL23 could be mediated directly through transcriptional regulation independently of additional intermediate cytokines, or may be dependent on one or more cytokines whose secretion rate was not measured and that were hence not included in the network (see the definition of a direct interaction, Definition 1). We subsequently validated several model predictions using experimental data that was not used for model fitting (datapoints in black in Fig 14). As predicted by the model, the experimentally observed up-regulation of IL23 by IFN*γ* is not pre-dominantly driven by the down-regulation of IL10 by IFN*γ*. Thus, IL23 is regulated by both IL10 (negative effect), and IL1 and to a lesser extent IFN*γ*. The possibility of such modelling predictions, as well as the explicit examples presented here did not feature in Aschenbrenner and Quaranta et al. [35]. We note that apart from the biological findings, the accuracy of the model predictions also validates the chosen form of our equations, e.g. the multiplicative, instead of for instance additive effects of the various cytokines on each other’s secretion rates (Eq (1)).

In summary, we have developed a method to extract a minimal, weighted network from cytokine interaction data using ordinary differential equations and the Akaike information criterion (AIC). This method enables us to distinguish direct from indirect effects of cytokine concentration on the secretion rate of other cytokines, to determine the strength of the different cytokine interactions, and to predict the effects of untested (combinations of) cytokine inhibitors. In contrast to directed acyclic graphs (DAGs), our model equations can accommodate self-edges and 2-cycles, such as the negative effect of IL10 on IL1 and the positive effect of IL1 on IL10 observed in the experimental IL23 dataset [22]. We validated our method using synthetic datasets and applied our method to an experimental dataset on the regulation of IL23. In addition to the extracted network, as first presented in Aschenbrenner and Quaranta et al. [35], here we have presented a validation framework and explicitly tested and confirmed numerous model predictions using experimental data. In future work, our method could be applied to other cytokine interaction data sets that include data on cytokine (receptor) blockades. Our type of model, i.e. a system of ODEs, allows for an easy extension to incorporate features that are currently often ignored, such as extensive longitudinal data, maximum cytokine secretion rates, or the interaction between cytokine responses of different cell types, such as T cells and monocytes. Together with the Akaike information criterion, we have used minimisation of the negative log-likelihood to obtain our model parameters, though future work could explore the prospect of extracting and using parameter posterior distributions. A limitation associated with the use of ODEs is the risk of model misspecification, though the validation of modelling predictions presented here a posteriori justifies the choices made for this study. In future work, the dependency of the selected network on the model equations and chosen error model could be explored. A further limitation is the requirement to minimize the log-likelihood for each model fit. Because the optimization of the log-likelihood function becomes computationally resource intensive for a growing number of equations and parameters, and because the number of possible network configurations quickly increases for a growing number of edges, a limitation of our method is the limited number of cytokines that can be studied at a time, though it can accommodate current experimental data generation. In particular, our method is intended to explore the interactions of a relatively small number of cytokines, such as currently typically found in intracellular cytokine staining datasets. When the use of spectral flow cytometers becomes more widely adopted, datasets with measurements of >30 different cytokines will become increasingly common. In such cases, the feasibility of the application of our method will depend on the amount of computational power available, and on the structure of the data, including the number of statistically significant interactions that need to be considered.

## Supporting information

S1 Appendix Experimental methods. (PDF)

S2 Appendix Implementation and code availability. (PDF)

## References

[1] Almradi A, Hanzel J, Sedano R, Parker CE, Feagan BG, Ma C, et al. Clinical Trials of IL-12/IL-23 Inhibitors in Inflammatory Bowel Disease. BioDrugs. 2020;34(6):713–721. doi: 10.1007/s40259-020-00451-w 33105016

[2] Danese S, Vermeire S, Hellstern P, Panaccione R, Rogler G, Fraser G, et al. Randomised trial and open-label extension study of an anti-interleukin-6 antibody in Crohn’s disease (ANDANTE I and II). Gut. 2019;68(1):40–48. doi: 10.1136/gutjnl-2017-314562 29247068 PMC6839832

[3] Friedrich M, Pohin M, Powrie F. Cytokine networks in the pathophysiology of inflammatory bowel disease. Immunity. 2019;50(4):992–1006. doi: 10.1016/j.immuni.2019.03.017 30995511

[4] Honap S, Meade S, Ibraheim H, Irving PM, Jones MP, Samaan MA. Effectiveness and Safety of Ustekinumab in Inflammatory Bowel Disease: A Systematic Review and Meta-Analysis. Digestive Diseases and Sciences. 2021. 33723700 10.1007/s10620-021-06932-4

[5] Baeten D, Baraliakos X, Braun J, Sieper J, Emery P, Van Der Heijde D, et al. Anti-interleukin-17A monoclonal antibody secukinumab in treatment of ankylosing spondylitis: A randomised, double-blind, placebo-controlled trial. The Lancet. 2013;382(9906):1705–1713. doi: 10.1016/S0140-6736(13)61134-4 24035250

[6] Bilsborough J, Targan SR, Snapper SB. Therapeutic targets in inflammatory bowel disease: current and future. The American Journal of Gastroenterology Supplements. 2016;3(3):27–37.

[7] Hueber W, Sands BE, Lewitzky S, Vandemeulebroecke M, Reinisch W, Higgins PDR, et al. Secukinumab, a human anti-IL-17A monoclonal antibody, for moderate to severe Crohn’s disease: Unexpected results of a randomised, double-blindplacebo- controlled trial. Gut. 2012;61(12):1693–1700. doi: 10.1136/gutjnl-2011-301668 22595313 PMC4902107

[8] McInnes IB, Sieper J, Braun J, Emery P, Van Der Heijde D, Isaacs JD, et al. Efficacy and safety of secukinumab, a fully human anti-interleukin-17A monoclonal antibody, in patients with moderate-to-severe psoriatic arthritis: A 24-week, randomised, double-blind, placebo-controlled, phase II proof-of-concept trial. Annals of the Rheumatic Diseases. 2014;73(2):349–356. doi: 10.1136/annrheumdis-2012-202646 23361084

[9] Roda G, Jharap B, Neeraj N, Colombel JF. Loss of Response to Anti-TNFs: Definition, Epidemiology, and Management. Clinical and Translational Gastroenterology. 2016;7(1):e135. doi: 10.1038/ctg.2015.63 26741065 PMC4737871

[10] Marino S, Myers A, Flynn JL, Kirschner DE. TNF and IL-10 are major factors in modulation of the phagocytic cell environment in lung and lymph node in tuberculosis: A next-generation two-compartmental model. Journal of Theoretical Biology. 2010;265(4):586–598. doi: 10.1016/j.jtbi.2010.05.012 20510249 PMC3150786

[11] Nagaraja S, Wallqvist A, Reifman J, Mitrophanov AY. Computational Approach To Characterize Causative Factors and Molecular Indicators of Chronic Wound Inflammation. Journal of Immunology. 2014;192(4):1824–1834. doi: 10.4049/jimmunol.1302481 24453259

[12] Anderson WD, Makadia HK, Greenhalgh AD, Schwaber JS, David S, Vadigepalli R. Computational modeling of cytokine signaling in microglia. Molecular BioSystems. 2015;11(12):3332–3346. doi: 10.1039/c5mb00488h 26440115 PMC5520540

[13] Wang Y, Yang T, Ma Y, Halade GV, Zhang J, Lindsey ML, et al. Mathematical modeling and stability analysis of macrophage activation in left ventricular remodeling post-myocardial infarction. BMC Genomics. 2012;13(Suppl 6):S21. doi: 10.1186/1471-2164-13-S6-S21 23134700 PMC3481436

[14] Morel PA, Lee REC, Faeder JR. Demystifying the cytokine network: Mathematical models point the way. Cytokine. 2017;98:115–123. doi: 10.1016/j.cyto.2016.11.013 27919524 PMC5457394

[15] Aikake H. A new look at the statistical model identification. IEEE Transactions on Automatic Control. 1974;AC-19:716–723. doi: 10.1109/TAC.1974.1100705

[16] Bozdogan H. Model selection and Akaike’s Information Criterion (AIC): The general theory and its analytical extensions. Psychometrika. 1987;52:345–370. doi: 10.1007/BF02294361

[17] Martinez-Sanchez ME, Huerta L, Alvarez-Buylla ER, Luján CV. Role of cytokine combinations on CD4+ T cell differentiation, partial polarization, and plasticity: Continuous network modeling approach. Frontiers in Physiology. 2018;9:877. doi: 10.3389/fphys.2018.00877 30127748 PMC6089340

[18] Martinez-Sanchez ME, Mendoza L, Villarreal C, Alvarez-Buylla ER. A minimal regulatory network of extrinsic and intrinsic factors recovers observed patterns of CD4+ T cell differentiation and plasticity. PLoS Computational Biology. 2015;11(6):e1004324. doi: 10.1371/journal.pcbi.1004324 26090929 PMC4475012

[19] Farhangmehr F, Maurya MR, Tartakovsky DM, Subramaniam S. Information theoretic approach to complex biological network reconstruction: Application to cytokine release in RAW 264.7 macrophages. BMC Systems Biology. 2014;8(1):77. doi: 10.1186/1752-0509-8-77 24964861 PMC4094931

[20] Field SL, Dasgupta T, Cummings M, Savage RS, Adebayo J, McSara H, et al. Bayesian modeling suggests that IL-12 (p40), IL-13 and MCP-1 drive murine cytokine networks in vivo. BMC Systems Biology. 2015;9(1):76. doi: 10.1186/s12918-015-0226-3 26553024 PMC4640223

[21] Margolin AA, Nemenman I, Basso K, Wiggins C, Stolovitzky G, Favera RD, et al. ARACNE: An algorithm for the reconstruction of gene regulatory networks in a mammalian cellular context. BMC Bioinformatics. 2006;7(S.1):S7. doi: 10.1186/1471-2105-7-S1-S7 16723010 PMC1810318

[22] Markowetz F, Spang R. Inferring cellular networks—A review. BMC Bioinformatics. 2007;8(Suppl. 6):S5. doi: 10.1186/1471-2105-8-S6-S5 17903286 PMC1995541

[23] Xue J, Schmidt SVV, Sander J, Draffehn A, Krebs W, Quester I, et al. Transcriptome-based network analysis reveals a spectrum model of human macrophage activation. Immunity. 2014;40(2):274–288. doi: 10.1016/j.immuni.2014.01.006 24530056 PMC3991396

[24] Rice JJ, Tu Y, Stolovitzky G. Reconstructing biological networks using conditional correlation analysis. Bioinformatics. 2005;21(6):765–773. doi: 10.1093/bioinformatics/bti064 15486043

[25] Magnusson R, Mariotti GP, Köpsén M, Lövfors W, Gawel DR, Jörnsten R, et al. LASSIM—A network inference toolbox for genome-wide mechanistic modeling. PLOS Computational Biology. 2017. doi: 10.1371/journal.pcbi.1005608 28640810 PMC5501685

[26] Bonneau R, Reiss DJ, Shannon P, Facciotti M, Hood L, Baliga NS, et al. The Inferelator: an algorithm for learning parsimonious regulatory networks from systems-biology data sets de novo. Genome Biology. 2006;7(5, R36). doi: 10.1186/gb-2006-7-5-r36 16686963 PMC1779511

[27] Nyman E, Stein RR, Jing X, Wang W, Marks B, Zervantonakis IK, et al. Perturbation biology links temporal protein changes to drug responses in a melanoma cell line. PLOS Computational Biology. 2020. doi: 10.1371/journal.pcbi.1007909 32667922 PMC7384681

[28] De Groote D, Zangerle PF, Gevaert Y, Fassotte MF, Beguin Y, Noizat-Pirenne F, et al. Direct stimulation of cytokines (IL-1*β*, TNF-*α*, IL-6, IL-2, IFN-*γ* and GM-CSF) in whole blood. I. Comparison with isolated PBMC stimulation. Cytokine. 1992;4(3):239–248. doi: 10.1016/1043-4666(92)90062-V 1498259

[29] Adachi O, Nakano A, Sato O, Kawamoto S, Tahara H, Toyoda N, et al. Gene tranfer of Fc-fusion cytokine by in vivo electroporation: Application to gene therapy for viral myocarditis. Gene Therapy. 2002;9(9):577–583. doi: 10.1038/sj.gt.3301691 11973633

[30] Raue A, Schilling M, Bachmann J, Matteson A, Schelke M, Kaschek D, et al. Lessons learned from quantitative dynamical modeling in systems biology. PLoS ONE. 2013;8(9):e74335. doi: 10.1371/journal.pone.0074335 24098642 PMC3787051

[31] Benjamini Y, Hochberg Y. Controlling the false discovery rate: a practical and powerful approach to multiple testing. Journal of the Royal Statistical Society: Series B (Methodological). 1995;57(1):289–300.

[32] Raue A, Kreutz C, Maiwald T, Bachmann J, Schilling M, Klingmüller U, et al. Structural and practical identifiability analysis of partially observed dynamical models by exploiting the profile likelihood. Bioinformatics. 2009;25(15):1923–1929. doi: 10.1093/bioinformatics/btp358 19505944

[33] Raue A, Steiert B, Schelker M, Kreutz C, Maiwald T, Hass H, et al. Data2Dynamics: a modeling environment tailored to parameter estimation in dynamical systems. Bioinformatics. 2015;31(21):3558–3560. doi: 10.1093/bioinformatics/btv405 26142188

[34] Kreutz C, Raue A, Timmer J. Likelihood based observability analysis and confidence intervals for predictions of dynamic models. BMC Systems Biology. 2012;6:120. Web. doi: 10.1186/1752-0509-6-120 22947028 PMC3490710

[35] Aschenbrenner D, Quaranta M, Banerjee S, Ilott N, Jansen J, Steere B, et al. Deconvolution of monocyte responses in inflammatory bowel disease reveals an IL-1 cytokine network that regulates IL-23 in genetic and acquired IL-10 resistance. Gut. 2020;(Web). 10.1136/gutjnl-2020-321731 PMC8108288 33037057

[36] Frucht DM, Fukao T, Bogdan C, Schindler H, O’Shea JJ, Koyasu S. IFN-*γ* production by antigen-presenting cells: Mechanisms emerge. Trends in Immunology. 2001;22(10):556–560. doi: 10.1016/S1471-4906(01)02005-1 11574279

[37] Boehm U, Klamp T, Groot M, Howard JC. Cellular responses to interferon-gamma. Annu Rev Immunol. 1997;15:749–95. 9143706 10.1146/annurev.immunol.15.1.749

[38] Castro F, Cardoso AP, Gonçalves RM, Serre K, Oliveira MJ. Crossroads of Tumor Immune Surveillance or Evasion. Front Immunol. 2018 May 4;9:847. doi: 10.3389/fimmu.2018.00847 29780381 PMC5945880

[39] Monaco G, Lee B, Xu W, Mustafah S, Hwang YY, Carré C, Burdin N, Visan L, Ceccarelli M, Poidinger M, Zippelius A, Pedro de Magalhães J, Larbi A. RNA-Seq Signatures Normalized by mRNA Abundance Allow Absolute Deconvolution of Human Immune Cell Types. Cell Rep. 2019 Feb 5;26(6):1627–1640.e7 doi: 10.1016/j.celrep.2019.01.041 30726743 PMC6367568

[40] Uhlig HH, Powrie F. Translating immunology into therapeutic concepts for inflammatory bowel disease. Annual Review of Immunology. 2018;36(9):755–781. doi: 10.1146/annurev-immunol-042617-053055 29677472

[41] Levy M, Arion A, Berrebi D, Cuisset L, Jeanne-Pasquier C, Bader-Meunier B, et al. Severe early-onset colitis revealing mevalonate kinase deficiency. Pediatrics. 2013;132(3):e779–e783. doi: 10.1542/peds.2012-3344 23979089

