# Supplementary material for: A method for the inference of cytokine interaction networks
Source: PLoS Comput Biol. 2022 Jun 22;18(6):e1010112. doi: 10.1371/journal.pcbi.1010112 (PMC9216621; doi:10.1371/journal.pcbi.1010112)
Supplement: S1 Appendix — (PDF) [file pcbi.1010112.s001.pdf]

A method for the inference of cytokine interaction networks
–
Supporting information

J.E. Jansen, D. Aschenbrenner, H.H. Uhlig, M.C. Coles and E.A. Gaffney

S1 Appendix: Experimental methods

All experimental data presented in this paper have been published before in [1], from which the following section was taken. Healthy donors were recruited via the Oxford gastrointestinal biobank (11/YH/0020 and 16/YH/0247). All healthy volunteers provided written informed consent. Whole blood was collected into EDTA-containing tubes. PBMC were purified using FicollPaque density gradient purification. The absolute number of cells was determined using a haemocytometer (Marienfeld). For the assays, 0.5×10^6 - 1×10^6 PBMC were cultured in 200 μl medium in duplicates in round bottom 96-well plates and exposed to ultrapure 200 ng/ml LPS (Enzo Life Sciences; Cat. # ALX-581-008) for 16 hours in complete RPMI with L-glutamine (Sigma) supplemented with 10 % human serum (Sigma; Cat.# H4522), non-essential amino acids (Gibco); 1 mM Sodium-Pyruvate (Gibco) and 100 U/ml penicillin and 10 μg/ml streptomycin (Sigma). The following neutralizing antibodies or receptor blocking antibodies were used (all 10 μg/ml): anti-IL-10R (Biolegend;clone: 3F9), anti-IL-1R1 (R&D; Cat.# AF269; polyclonal Goat IgG), anti-IL-1a/IL-1F1 (R&D; Cat.# AF-200-NA; polyclonal Goat IgG), anti-IL-1b/IL-1F2 (R&D; Cat.# MAB201; clone: 8516), anti-IL-6R (Tocilizumab, ActemraÒ, Roche,), antiTNFa (Infliximab, REMICADEÒ, Janssen), anti-IL-10R (Biolegend; Cat.# 308807; clone: 3F9). Cells were stimulated in the presence of brefeldin A (BFA, 10 μg/ml, all from Sigma) for the final 4 hours of cell culture. Cells were fixed and permeabilised with Cytofix/Cytoperm (BD Biosciences) according to the manufacturer's instructions. To exclude dead cells from the analysis, cells were stained prior to fixation using Fixable Viability Dye eFluor 780 (eBioscience). Following fluorophore-conjugated anti-cytokine antibodies were used for analysis (supplier; clone): anti-IL-1a (Biolegend; 364-3B3-14), anti-IL-1b (Biolegend; H1b98), anti-IL-6 (eBioscience; MQ2-13A5), anti-IL-10 (Biolegend; JES3-9D7), anti-IL-23p19 (eBioscience; 23dcdp), anti-TNF (Biolegend: MAb11). For the detection of IL-12p40 the combination of biotin-conjugated anti-IL-12p40 (BD Biosciences; C8.6) and streptavidin STV-CF594 (BD Biosciences; Cat.# 562318) were used. Data were acquired on a LSRII flow cytometer (BD Biosciences).

References

1. Aschenbrenner D, Quaranta M, Banerjee S, Ilott N, Jansen J, Steere B, et al. Deconvolution of monocyte responses in inflammatory bowel disease reveals an IL-1 cytokine network that regulates IL-23 in genetic and acquired IL-10 resistance. Gut. 2020;(Web). doi:10.1136/gutjnl-2020-321731.
