# Supplementary material for: A method for the inference of cytokine interaction networks
Source: PLoS Comput Biol. 2022 Jun 22;18(6):e1010112. doi: 10.1371/journal.pcbi.1010112 (PMC9216621; doi:10.1371/journal.pcbi.1010112)
Supplement: S2 Appendix — (PDF) [file pcbi.1010112.s002.pdf]

A method for the inference of cytokine interaction networks
–
Supporting information

J.E. Jansen, D. Aschenbrenner, H.H. Uhlig, M.C. Coles and E.A. Gaffney

S2 Appendix: Implementation and code availability

Our method depends on the global minimization of the log-likelihood (Eq (3)), which is a non-trivial task. Throughout this paper (Sections "Validation" and "Application to a dataset on IL23 regulation"), we used a deterministic trust-region approach combined with a multi-start strategy for this problem, as implemented in the Matlab-based modelling environment 'Data2Dynamics' [1]. This approach has been found to be the most accurate and the fastest in a systematic comparison of optimization algorithms used for this task, including twelve stochastic algorithms such as a standard evolutionary optimizer, an evolutionary optimizer with covariance matrix adaption (CMA-ES), and particle swarm optimization, and a hybrid scatter search algorithm [2]. We note that our method is independent of this choice, and other optimization algorithms could be used instead. Briefly, we implemented the system of equations (1) in the Matlab based modelling environment 'Data2Dynamics' [1]. For each estimate of $D = \min_{\boldsymbol{\theta}} -2\log\left(\mathcal{L}(\boldsymbol{\theta})\right)$, we ran the trust-region algorithm multiple times, with different initial parameter value guesses, where each run returns the value of D after convergence of the algorithm to a local optimum. We will discuss below how we select a value from these estimates that we assume represents a global minimum, i.e. the true value of D. For the test model datasets, the initial guesses for each parameter s_i, $\alpha_{u,i}$ and $\beta_{v,i}$ were sampled from a log-10 transformed uniform interval, with lower bound 10^{-2} and upper bound 10^{1}. For the IL23 model, the initial guesses were sampled with lower (upper) bounds on s_i of $10^{-1.5}$ (10^{0}) and lower (upper) bounds on $\alpha_{u,i}/\beta_{v,i}$ of 10^{-2} (10^{1}). Latin-hypercube sampling (LHS) from the initial guess-sampling intervals was used to obtain a set 51 of initial guesses. Given the number of model parameters, we emphasize this corresponds to a very coarse coverage of the different regions in parameter space. The regular shape of all profile likelihood profiles of our five cytokine test model (Fig 9), the validation of the coverage of the corresponding confidence intervals (Fig 11 and Fig 12), and the number of minima returned for each run of the algorithm (see below), serve to motivate our assumption that the 51 initial guesses are nonetheless sufficient to obtain an estimate of the global minimum. We set a lower bound on the parameter space for all fitted parameters of 10^{-20} and an upper bound of 10^{5}. We note the initial guess-sampling intervals were set much smaller than this wide range. When we decreased the lower bound of the initial guess-sampling intervals to 10^{-20}, we observed that the optimization algorithm returned a large number of local minima with one or more fitted parameter values smaller than 10^{-16}. We show an example for the IL23 model configuration with all significant edges, for 10,000 initial guesses with a lower bound of the initial guess-sampling interval of 10^{-20} (Fig A, left). The minimal D

returned by the algorithm is shown as a red dot. When we increased the lower bound of the interval from which the initial guesses were sampled to 10^{-2}, the number of returned local minima deceased. We note that all minima, other than the minimum indicated with a red dot, effectively correspond to cases where one or more edges are removed from the model, as the value of at least one of the parameter values is smaller than machine precision. The first reason to constrain the initial guess-sampling intervals is to decrease the number of times the algorithm gets trapped at such local minima, which are untrustworthy due to the manipulations of parameter values that are smaller than machine precision. A second reason to constrain the initial guess-sampling intervals is that, for a given number of initial guesses, we can sample more densely from the region in parameter space where we expect to find a genuine, physiological minimum.

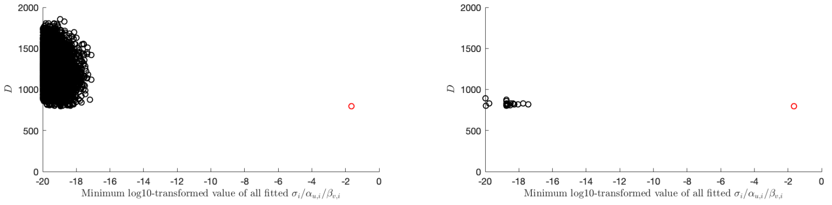

Fig A. We reduce the number of local minima returned by the optimization algorithm by increasing the lower bound of the initial guess-sampling interval from 10^{-20} (left) to 10^{-2} (right). Values of D returned by the optimization algorithm against the minimal value of each fitted parameter corresponding to this local minimum. We ran the optimization algorithm with a 10.000 different initial guesses for our parameter values. The minimal D is shown as a red dot (at the right-hand side of the figure). All other (local) minima are shown as black dots (at the left-hand side of the figure). We note that these local minima effectively correspond to cases were one or more edges are removed from the model, as the value of at least one of the parameter values is smaller than machine precision.

All parameter values $\alpha_{u,i}$ and $\beta_{v,i}$ corresponding to non-existing edges in the modelled network configuration were fixed to zero and not estimated. For all estimates of D presented, we initially ran the deterministic trust-region algorithm with 51 different initial guesses. When an initial guess did not return a local optimum, for instance when model evaluation was not feasible for this combination of parameter values, we resampled parameter values until a feasible initial guess was found. Infeasible initial guesses occur when multiple upregulatory edges drive the expression of one of the cytokines to an infeasibly large value value (10^{20} was used in practice). When more than one local minimum was returned by the algorithm, we determined the smallest parameter value for each returned minimum, out of all fitted parameter values. We distinguish two cases. When this smallest parameter was smaller than 10^{-16} for all minima, excluding the minimum with the smallest D, such local minima represent cases where the algorithm gets trapped in a region with a very small parameter value, corresponding to a subconfiguration of the model configuration of interest (black dots, Fig A). We assume the minimum with the lowest D represents the global minimum (red dot, Fig A) and ignore all other minima, each of which contain a parameter value smaller than 10^{-16}. Thus, the minimum with the lowest D is the selected minimum that gets returned by the algorithm. We note that when the model contains edges that do not improve the model fit, not a single minimum with all with parameter values larger than 10^{-16} is returned by the algorithm. In such cases the algorithm also selects the minimum with the lowest D out of all returned minima. When multiple minima are found, each with all fitted parameter values larger than 10^{-16}, these represent distinct

local minima for the network configuration of interest. This is the second case we distinguish. Based on the number of local minima returned, 'Data2Dynamics' gives estimates of the real number of local minima using a number of non-parametric methods from the literature, such as the Jackknife1 and Chao2 methods [3,4]. E.g., the Jackknife 1 method estimates the true number of minima M_{Jack_1} as $M_{Jack_1} = M_{observed} + Q_1(m-1)/(m)$, with $M_{observed}$ the number of observed minima, Q_1 the number of minima that are observed once, and m the number of initial guesses. When multiple minima are observed, all with parameter values larger than 10^{-16}, we could increase the number of initial guesses until each minimum is observed at least twice, such that $M_{Jack_1} = M_{observed}$. We note we did not have to increase the number of initial guesses in this way for the models presented in this paper (see below). 'Data2Dynamics' considers two minima distinct when the difference between their respective value of D is larger than 0.01. The algorithm selects the minimum with the lowest D out of all returned minima.

For the IL23 model, the D was computed for 60 different model configurations, each with 51 runs of the trust-region algorithm, with different initial parameter value guesses sampled used Latin-hypercube sampling. In 49 out of 60 of these cases, all 51 runs of the trust-region algorithm returned the same optimum. In 11 out of 60 of these cases, 50 runs returned the same optimum $\bar{D}$ and 1 run returned a local optimum, larger than $\bar{D}$, containing a parameter value smaller than 10^{-16}. For the five cytokine test model, the D was computed for 120 different model configurations. In 30 out of 120 of these cases, all 51 initial runs returned the same optimum. In 89 out of 120 of these cases, multiple local optima were returned, larger than $\bar{D}$, containing a parameter value smaller than 10^{-16}. For a single case, two local minima were returned, both with all parameter values larger than 10^{-16}. Both minima were observed multiple times (22 and 29 times). Out of the two minima we picked the minimum with the smallest D. The smallest edge parameter value for this minimum was 10^{-2}. The other minimum, with a larger D, contained an edge parameter with a value smaller than 10^{-8}. We note that when we would have encountered a case where a minimum with all parameter values larger than 10^{-16} was observed only once out of the 51 runs of the optimization algorithm, we would have increased the number of initial guesses until this minimum was observed at least twice and then selected the minimum with the lowest D out of all returned minima. We did not encounter such a case. We emphasize that we cannot proof that the minima selected by the algorithm are indeed global optima, but as motivated previously we will assume for the remainder of the paper that all minima selected by the algorithm are global. Running the model selection algorithm took 6.1 hours and 0.25 hours for the five cytokine test model and IL23 model respectively, on a Macbook Pro laptop with a 2.3 GHz Intel Core i5 processor.

The Matlab based modelling environment 'Data2Dynamics' allows for an investigation of the identifiability of the parameters of the implemented model, using the Profile Likelihood Approach [5]. Confidence intervals for model predictions can be obtained by the Prediction Profile likelihood approach by Kreutz et al. [6]. The 'Data2Dynamics' toolbox does not calculate prediction profiles directly, but infers them using the Validation Profile likelihood approach. We refer to the 'Data2Dynamics' documentation and [6] for further details on the implementation of this approach.

Our implementation of the model selection algorithm is available at `http://github.com/Joanneke-Jansen/cytokine-network-inference`. We included an application to our IL23 dataset, as an example of the model selection process (Section "Application to a dataset on IL23 regulation").

References

1. Raue A, Steiert B, Schelker M, Kreutz C, Maiwald T, Hass H, et al. Data2Dynamics: a modeling environment tailored to parameter estimation in dynamical systems. Bioinformatics. 2015;31(21):3558–3560. doi:10.1093/bioinformatics/btv405.

2. Raue A, Schilling M, Bachmann J, Matteson A, Schelke M, Kaschek D, et al. Lessons learned from quantitative dynamical modeling in systems biology. PLoS ONE. 2013;8(9):e74335. doi:10.1371/journal.pone.0074335.

3. Colwell RK. EstimateS: Statistical estimation of species richness and shared species from samples. Version 9. http://viceroy.eeb.uconn.edu/estimates/; 2013.

4. Magurran AE. Measuring biological diversity. Ebook central. Malden, Ma.: Blackwell Pub.; 2004.

5. Raue A, Kreutz C, Maiwald T, Bachmann J, Schilling M, Klingmüller U, et al. Structural and practical identifiability analysis of partially observed dynamical models by exploiting the profile likelihood. Bioinformatics. 2009;25(15):1923–1929. doi:10.1093/bioinformatics/btp358.

6. Kreutz C, Raue A, Timmer J. Likelihood based observability analysis and confidence intervals for predictions of dynamic models. BMC Systems Biology. 2012;6:120. Web. doi:10.1186/1752-0509-6-120.
